# Antagonistic Regulation of Apoptosis and Differentiation by the Cut Transcription Factor Represents a Tumor-Suppressing Mechanism in *Drosophila*

**DOI:** 10.1371/journal.pgen.1002582

**Published:** 2012-03-15

**Authors:** Zongzhao Zhai, Nati Ha, Fani Papagiannouli, Anne Hamacher-Brady, Nathan Brady, Sebastian Sorge, Daniela Bezdan, Ingrid Lohmann

**Affiliations:** 1 CellNetworks–Cluster of Excellence, Centre for Organismal Studies (COS) Heidelberg, University of Heidelberg, Heidelberg, Germany; 2 German Cancer Research Center (DKFZ), Heidelberg, Germany; 3 Max Planck Institute for Developmental Biology, Tübingen, Germany; University of California San Francisco, United States of America

## Abstract

Apoptosis is essential to prevent oncogenic transformation by triggering self-destruction of harmful cells, including those unable to differentiate. However, the mechanisms linking impaired cell differentiation and apoptosis during development and disease are not well understood. Here we report that the *Drosophila* transcription factor Cut coordinately controls differentiation and repression of apoptosis via direct regulation of the pro-apoptotic gene *reaper*. We also demonstrate that this regulatory circuit acts in diverse cell lineages to remove uncommitted precursor cells *in status nascendi* and thereby interferes with their potential to develop into cancer cells. Consistent with the role of Cut homologues in controlling cell death in vertebrates, we find repression of apoptosis regulators by Cux1 in human cancer cells. Finally, we present evidence that suggests that other lineage-restricted specification factors employ a similar mechanism to put the brakes on the oncogenic process.

## Author Summary

Apoptosis is a highly conserved cellular function to remove excessive or unstable cells in diverse developmental processes and disease-responses. An important example is the elimination of cells unable to differentiate, which have the potential to generate tumors. Despite the significance of this process, the mechanisms coupling loss of differentiation and apoptosis have remained elusive. Using cell-type specification in *Drosophila* as a model, we now identify a conserved regulatory logic that underlies cell-type specific removal of uncommitted cells by apoptosis. We find that the transcription factor Cut activates differentiation, while it simultaneously represses cell death via the direct regulation of a pro-apoptotic gene. We show that this regulatory interaction occurs in many diverse cell types and is essential for normal development. Using *in vivo Drosophila* cancer models, we demonstrate that apoptosis activation in differentiation-compromised cells is an immediate-early cancer prevention mechanism. Importantly, we show that this type of regulatory wiring is also found in vertebrates and that other cell-type specification factors might employ a similar mechanism for tumor suppression. Thus, our findings suggest that the coupling of differentiation and apoptosis by individual transcription factors is a widely used and evolutionarily conserved cancer prevention module, which is hard-wired into the developmental program.

## Introduction

It has been a long-standing paradigm that impaired cell fate commitment is a key initiator of cancer development [1], [2], since cancer cells display reduced differentiation properties compared to normal cells, while tumor formation can be suppressed by inducing the terminal cell fate in cancer cells [3]. The molecular basis of the interplay between cell differentiation and cancer has only recently been established. Bossuyt and colleagues (2009) demonstrated that loss of the proneural transcription factor Atonal not only leads to a loss of differentiated eye tissue but also promotes tumor formation and progression in this tissue context [4]. Thus, their work provided evidence that the maintenance of a differentiated state, which is critically controlled by a cell-type specification factor, is one crucial aspect to prevent the oncogenic process, whereas loss of this master regulator, together with other mutations creating a sensitized background, leads to the initiation of tumorigenesis. In order to evade tumor development, organisms have evolved potent mechanisms to protect themselves from the effects of mutations in their soma [5]. Programmed cell death, or apoptosis, plays a crucial role in removing abnormal cells, which could develop into tumors. This is supported by the observation that most types of cancers are associated with genetic alterations that deactivate this rescue pathway, most commonly via up-regulation of anti-apoptotic genes [6].

Since loss of terminal differentiation and the inability to activate apoptosis are crucial steps in cancer development, the existence of regulatory mechanisms preventing the accumulation of cells harboring mutations in both pathways seems essential for the survival of multi-cellular organisms. Consistently, mutations in differentiation genes very often result in the activation of the programmed cell death machinery [7], [8]. However, the mechanisms linking loss of differentiation and induction of apoptosis, which is crucial for the prevention of tumor formation, are still missing. Here we have used the *Drosophila* posterior spiracle (PS) as a model to analyze the interplay of differentiation and apoptosis at the mechanistic level. By studying the morphogenesis of this organ, we have identified a hard-wired program through which the cell-type specifying transcription factor Cut (Ct) controls in a subset of PS cells, the filzkörper cells, initiation of differentiation and simultaneous repression of apoptosis via the direct transcriptional regulation of the pro-apoptotic gene *rpr*. Using two well-established *Drosophila in vivo* eye cancer models, we demonstrate that this regulatory circuit instructed by the transcription factor Ct is a very potent mechanism to prevent and/or reduce tumor growth, as it allows the lineage-specific removal of abnormal cells at the time of their genesis. Moreover, our data show that a related regulatory wiring is used in vertebrates and that other cell-type specification factors might employ a similar mechanism for tumor suppression, thus suggesting that the coupling of differentiation and apoptosis by individual transcription factors is a widely used and evolutionary conserved cancer prevention module, which is hard-wired into the developmental program.

## Results

### Cut inhibits *rpr* expression and induction of apoptosis in the PS

**Figure 1:**
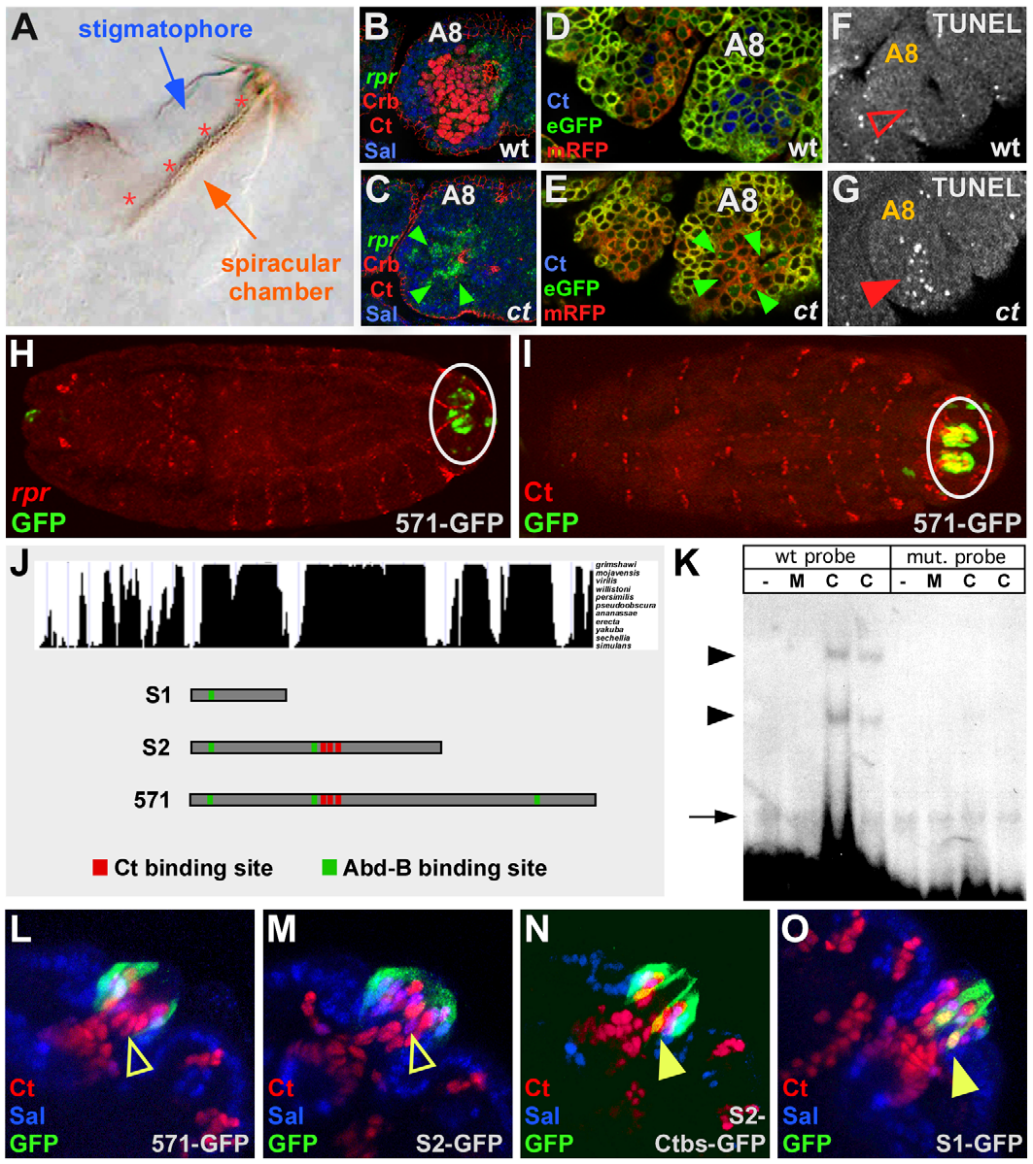
Cut directly represses *rpr* and apoptosis in the PS primordium. (A) Posterior spiracle (PS) of a 1^st^ instar wild-type *Drosophila* larva. The filzkörper is highlighted by red asterisks. (B, C) *rpr* mRNA (green) expression in stage 11 wild-type (B) and *ct* mutant (C) embryos. Spalt (Sal) protein (blue) labels stigmatophore precursor cells, Cut (Ct) protein (red, nuclear) marks spiracular chamber and filzkörper precursor cells and the apical membrane marker Crb (red) outlines the cells. Small, green arrows in (C) mark *rpr* positive spiracular chamber and filzkörper precursor cells in the eighth abdominal segment (A8) of *ct* mutant embryos. (D, E) Over-expression of the apoptosis sensor UAS-*Apoliner* using the *arm*-GAL4 driver in stage 11 wild-type (D) and *ct* mutant (E) embryos. Small, green arrows in (E) mark apoptotic cells in PS precursor cells (A8) of *ct* mutant embryos. (F, G) TUNEL stainings in wild-type (F) and *ct* mutant (G) embryos. Closed arrowhead in (G) marks TUNEL-positive cells in *ct* mutants, which are absent in wild-type embryos (F). (H, I) Co-localization of GFP protein and *rpr* mRNA (H) or Cut protein (I) in stage 15 *rpr*-HRE-571 embryos. White circles mark the PS primordium. (J) Top: conservation blot of *rpr*-HRE-571 genomic region obtained from the UCSC genome browser (http://genome.ucsc.edu/). Species used for generating blot are also shown in Figure S2A. Bottom: diagram of the *rpr*-HRE-571 deletion constructs tested. (K) EMSA using S2 sub-fragment with Ct binding sites either in wild-type (wt probe) or mutated (mut. probe) version and no protein (−), purified MBP protein (M), and purified Cut-MBP fusion protein consisting of the Cut repeat 3 and the Cut homeodomain (C). The black arrowheads indicate the specific DNA-protein complexes. Loading of equal amounts of labeled wild-type and mutated oligonucleotides is illustrated by formation of comparable amounts of unspecific DNA-protein complexes (black arrow). (L–O) Reporter gene expression in the PS of stage 15 embryos driven by the fragments described above. In the S2-Ctbs-GFP, line Ct binding sites within the *rpr*-HRE-571-S2 fragment are mutated. Spalt (Sal) and Cut (Ct) proteins label stigmatophore (blue) or spiracular chamber and filzkörper cells (red). Closed, yellow arrowheads in (N) and (M) mark reporter gene expression in filzkörper cells, whereas open, yellow arrowheads in (L) and (M) mark missing GFP expression.

**Figure 2:**
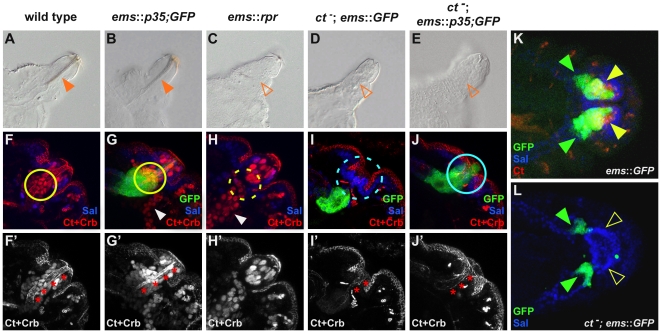
Cut-dependent repression of apoptosis is required for cell survival and differentiation. (A–E) Cuticle preparations of the different genotypes with focus on the PS of 1^st^ instar *Drosophila* larvae. Closed, orange arrowheads in (A) and (B) mark the filzkörper, whereas open, orange arrowheads in (C, D and E) indicate the absence of this structure in the respective genotypes. (F–L) Labeling of the different parts of the PS primordium of stage 15 embryos in the different genetic backgrounds using the filzkörper marker Ct (red, nuclear), the stigmatophore marker Sal (blue) and the apical membrane marker Crb (red). In (G, I, J, K and L) GFP expression (green) driven by the *ems*-GAL4 driver is shown in the different genetic backgrounds. Red asterisks in (F′–J′) mark the invaginated cells of the future filzkörper. Closed, yellow circles in (F) and (G) mark Ct-positive, invaginated filzkörper precursor cells, dashed yellow circle in (H) indicates the absence of these cells. Dashed, light blue circle in (I) highlights the absence of GFP-positive cells, whereas closed, light blue circle in (J) mark the presence of these cells. Note that some cells expressing *GFP* under the control of the *ems*-GAL4 driver invaginate deeper than the Ct expressing cells, thus they are still present in *ct^db7^* mutant embryos, indicated by closed, green arrowheads in (K) and (L). Closed, yellow arrowhead in (K) marks Ct and GFP-positive cells in *ems*::*GFP* embryos. Open, yellow arrowhead in (L) highlights the absence of these cells in *ct* mutant embryos. In (A) to (J′) lateral views, in (K) and (L) dorsal views of embryos are shown.

The PS connects the *Drosophila* respiratory system to the environment and consists of an internal tube, the spiracular chamber with a refractile filter, the filzkörper, which is specified by the transcription factor Ct, and an external protrusion in which the spiracular chamber is located, the stigmatophore, which is under the control of the transcription factor Spalt (Sal) (Figure 1A; Figure S1A–S1D) [9]. In 1^st^ instar *ct* mutant larvae filzkörper cells are not detectable (Figure 2A, 2D; Figure S5A, S5B), which *a priori* suggests that Ct is primarily required for the specification of the filzkörper cell fate. However, due to the fact that Ct has also been shown to regulate programmed cell death [7], we assumed that filzkörper cells in *ct* mutant embryos could be completely missing due to the induction of apoptosis. To test this hypothesis, we analyzed the expression of all *Drosophila* pro-apoptotic genes, which revealed the specific repression of *reaper* (*rpr*) (Figure 1B, 1C; Figure S1E, S1F; Figure S2A, S2B) but not of *head involution defective* (*hid*), *grim* and *sickle* (*skl*) (Figure S1I–S1N) transcription by Ct in embryonic filzkörper precursor cells. Strikingly, we only observed *rpr* de-repression in Ct-positive filzkörper, but never in Ct-neighboring, Sal-positive stigmatophore precursor cells (Figure 1B, 1C; Figure S1E, S1F), evidencing the cell-autonomous regulation of *rpr* by Ct. Rpr binds to Inhibitor of Apoptosis Protein (IAP), thereby releasing inhibition of caspases and promoting apoptosis [10]. Consistently we could demonstrate enhanced cell death in *ct* deficient filzkörper precursor cells of stage 11 embryos using the genetically-encoded caspase reporter Apoliner [11] as well as TUNEL and Acridine Orange (AO) stainings (Figure 1D, 1E, 1F, 1G; Figure S2G, S2H). Thus, *rpr* de-repression is followed by apoptosis induction in *ct* mutant embryos. To study the interplay between cell-type specification and cell death at the mechanistic level, we identified conserved Ct-dependent regulatory regions in the *rpr* intergenic regions using computational methods. Due to the principal requirement of the Hox transcription factor Abdominal-B (Abd-B) for PS development [9], we searched for clusters of binding sites for Abd-B and Ct and found a highly conserved 571 bp DNA element close to the *rpr* coding region, which we termed *rpr*-HRE-571 (Figure S4). Sequence-specific interaction of recombinant Ct protein with part of the enhancer module, the S2 sub-fragment, was detected by electrophoretic mobility shift assays (Figure 1K; Figure S1G). Immunostainings revealed *rpr*-HRE-571-GFP activity solely in stigmatophore (Figure 1I, 1L; Figure S3A, S3E, S3I, S3M) but not in Ct-positive filzkörper precursor cells (Figure 1L; Figure S3A, S3E, S3I, S3M), that do not express *rpr* (Figure 1B, 1H; Figure S1E). To validate the *in vivo* interaction of Ct with the identified enhancer module, we interfered with Ct-enhancer interaction in two ways: we mutated Ct binding sites within the *rpr*-HRE-571-S2 fragment (Figure 1J), a truncated module with identical activity as the *rpr*-HRE-571 enhancer (Figure 1M; Figure S3B, S3F, S3J, S3N), and eliminated all three sites in a small deletion version of the *rpr*-HRE-571 enhancer, termed *rpr*-HRE-571-S1 (Figure 1J). In both cases, GFP expression was ectopically activated in Ct-positive filzkörper precursor cells (Figure 1N, 1O; Figure S3C, S3D, S3G, S3H, S3K, S3L, S3O, S3P). These experiments demonstrated that Ct directly represses *rpr* transcription and thus apoptosis in the filzkörper precursor cells of the PS in a cell-autonomous manner by interacting with a small enhancer module located in the *rpr* intergenic region.

### Repression of apoptosis by Ct is required for differentiation of filzkörper cells

Since our result showed that filzkörper cells are very efficiently eliminated by apoptosis in the absence of Ct function, we next asked whether Ct primarily acts as a repressor of programmed cell death or whether this factor is also required for the differentiation of filzkörper cells. To this end, we analyzed *ct* deficient cells, which were kept alive by expressing the caspase inhibitor p35 [12] in *ct* mutant embryos using the PS-specific driver *ems*-GAL4 [13]. In order to follow the cells normally under the control of Ct, these cells were GFP-labeled using the same driver, which is active only in a subset of Ct-expressing cells (Figure 2G, 2K). Our experiments revealed that Ct- and GFP-positive filzkörper cells found in the wild-type situation (Figure 2A, 2F, 2F′, 2B, 2G, 2G′, 2K; Figure S5A, S5D, S5G) are eliminated in *ct* mutant embryos (Figure 2D, 2I, 2I′, 2L; Figure S5B, S5E, S5H), whereas they remained viable when apoptosis is blocked (*ct^−^*; *ems*::*p35*) (Figure 2E, 2J, 2J). However, these *ct* deficient, undead cells had developmental defects, as they did not properly invaginate and did not acquire their terminal cell fate as indicated by reduced expression of the apical cell polarity marker Crumbs (Crb) and the cell adhesion molecule DE-Cadherin (Figure 2F′, 2J′; Figure S5D, S5F, S5G, S5I). Consistently, these cells never adopted a filzkörper cell fate (Figure 2E; Figure S5C). These defects were a consequence of blocking cell death in *ct* deficient, undifferentiated cells and were not due to a general response to the apoptosis inhibitor p35, as the filzkörper of *ems*::*p35* control embryos (Figure 2B, 2G, 2G′) was indistinguishable form those of wild-type embryos (Figure 2A, 2F, 2F′). Local activation of apoptosis was sufficient to induce cell death in filzkörper cells, as expression of a *rpr* transgene resulted in their elimination (Figure 2C, 2H, 2H′). Taken together, our results revealed that Ct carries out two functions during PS morphogenesis: it allows the survival of uncommitted precursor cells by the transcriptional repression of the pro-apoptotic gene *rpr* and subsequently it drives these cells into a filzkörper-specific cell fate.

### Simultaneous regulation of apoptosis and cell fate commitment is a general function of Cut

**Figure 3:**
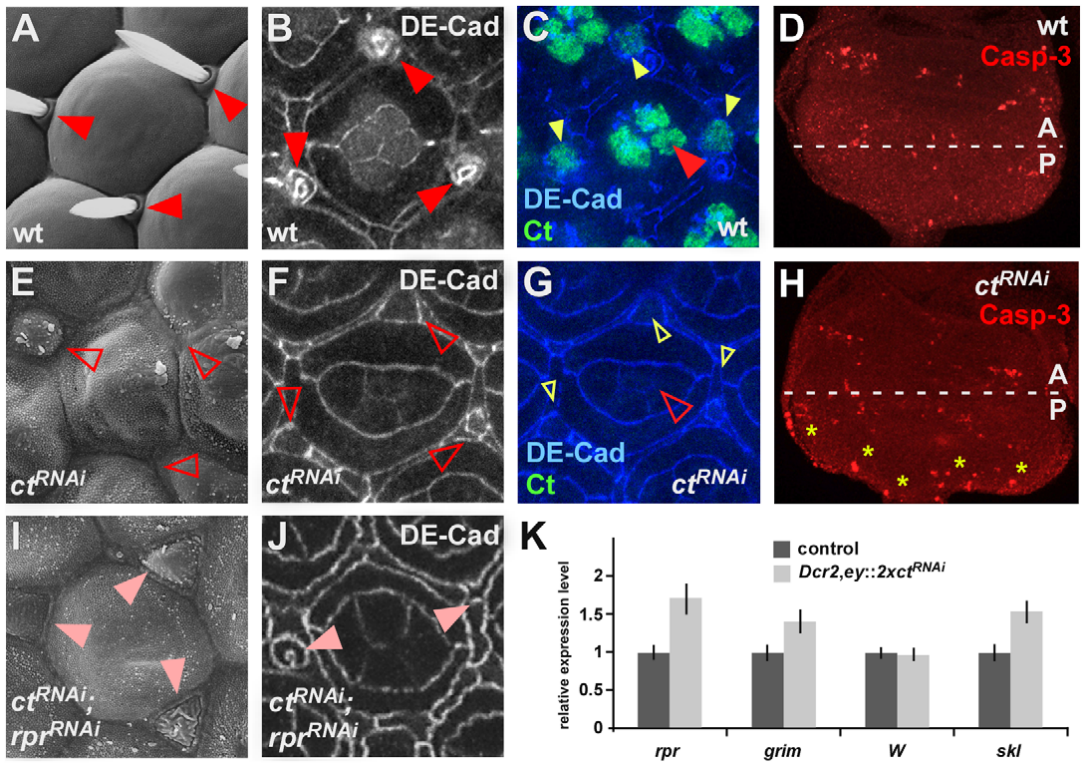
General function of Ct in apoptosis repression and induction of differentiation. (A, E, I) Scanning electron micrographs of individual ommatidia of adult *Drosophila* fly eyes with indicated genotypes are shown. The closed, red arrowheads in (A) mark interommatidial bristles, the open, red arrowheads in (E) mark the absence of these structures. The closed, light red arrowheads in (I) indicate the presence of tissue that would normally develop into interommatidial bristles. (B, F, J) Projections of consecutive confocal sections of one ommatidium of 50 h pupal retinas labeled with DE-Cadherin. Interommatidial bristles are marked by red, closed arrowheads in (B). Open arrowheads in (F) mark absence of DE-Cad, light-red arrowheads in (J) mark reduced DE-Cadherin levels in shaft cells of interommatidial bristles. (C, G) Projections of consecutive confocal sections of one ommatidium of 50 h pupal retinas of *GMR::lacZ* control (C) and *GMR::ct^RNAi^* flies (G). (D, H) Expression of the apoptosis marker Caspase-3 (Casp-3) in 3^rd^ instar eye-antennal discs of control *Dcr2; ey::lacZ* (D) and *Dcr2; ey::2xct^RNAi^* (H) animals. Yellow asterisks in (H) mark Casp-3 positive cells in *Dcr2; ey::2xct^RNAi^* eye imaginal discs. (K) Relative mRNA expression levels of *rpr*, *grim*, *Wrinkled* (*W*) and *sickle* (*skl*) in 3^rd^ instar eye-antennal discs of control *Dcr2; ey::lacZ* and *Dcr2; ey::2xct^RNAi^* animals.

**Figure 4:**
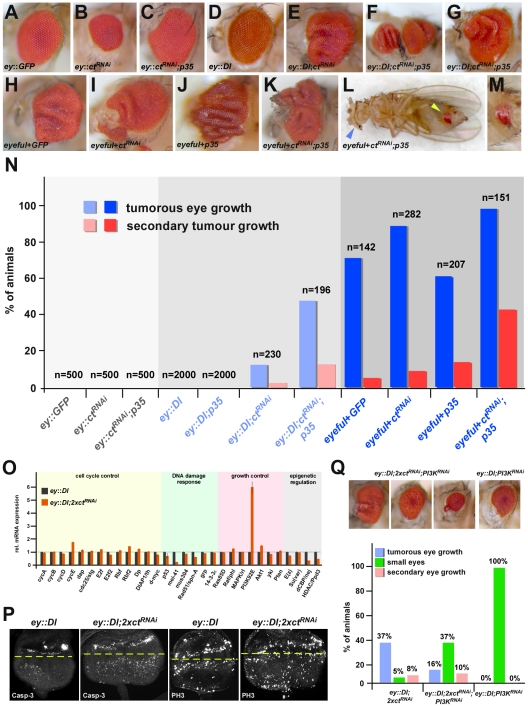
The Ct switch function represents a cancer prevention mechanism. (A–M) Adult compound eyes of the respective genotypes are shown. (L) *eyeful::ct^RNAi^; p35* flies show high frequency of long range metastasis (marked by yellow arrowhead), a close-up of which is shown in (M). Eyes of such *eyeful::ct^RNAi^; p35* flies show undifferentiated and overproliferated eye tissue (marked by light blue arrowhead). (N) Quantification of primary and secondary tumor formation in different genetic backgrounds. (O) Relative transcript levels of selected genes involved in cell cycle control, DNA damage response, growth control and epigenetic regulation in eye-antennal discs of 3^rd^ instar larvae of pre-oncogenic control animals (*ey::Dl*) and animals with reduced Ct activity (*ey::Dl;2xct^RNAi^*). (P) Expression of the apoptosis marker Caspase-3 (Casp-3) and the proliferation marker Phosphorylated histone H3 (PH3) in representative 3^rd^ instar eye-antennal discs of *ey::Dl* and *ey::Dl;2xct^RNAi^* animals. An increase in Casp-3 and PH3 positive cells is seen in the area below the dashed, yellow line highlighting the morphogenetic furrow. (Q) Top panel: representative pictures of eyes from *ey::Dl;PI3K^RNAi^* and *ey::Dl;2xct^RNAi^;PI3K^RNAi^* animals. Bottom panel: quantification of tumorous eye growth, secondary tumor growth and “small eye” phenotype in *ey::Dl;2xct^RNAi^* and *ey::Dl;2xct^RNAi^;PI3K^RNAi^* and *ey::Dl;PI3K^RNAi^* animals.

Ct is expressed in many different cell and tissue types [14], thus we tested the Ct switch function in diverse developmental contexts. Ct activity was eliminated in the *Drosophila* eye using RNAi (Figure 3C, 3G), resulting in an overall reduction of the eye size (Figure 4A, 4B), and a loss of interommatidial bristles (Figure 3A, 3E), which normally express Ct (Figure 3C). Consistently, expression of the bristle shaft progenitor marker DE-Cadherin [15] was lost in *GMR*::*ct^RNAi^* pupal retinas (Figure 3B, 3F). Expression of the apoptotic executor activated Caspase-3 was significantly increased in eye discs of *ey*::*ct^RNAi^* 3^rd^ instar larvae (Figure 3D, 3H), and a significant induction of *rpr* RNA levels was observed using quantitative Real Time-PCR (qRT-PCR) (Figure 3K). Co-expression of either the apoptosis inhibitor p35, which rescues the eye size (Figure 4C), or of a *rpr^RNAi^* construct along with the *ct^RNAi^* transgene resulted in a survival of *ct* deficient cells, as evidenced by the expression of the bristle shaft progenitor maker DE-Cadherin (Figure 3J). However, reminiscent to the phenotypes in the PS (Figure 2E, 2J′), these cells were unable to adopt their terminal fate due to the absence of the cell-specification factor Ct, and consequently fully differentiated interommatidial bristles were absent (Figure 3I). Similar results were obtained in other cell types specified by Ct (Figure S6), suggesting that the Ct-dependent switch between cell-type specification and programmed cell death is of general relevance.

### Simultaneous and antagonistic regulation of differentiation and apoptosis represents a cancer prevention mechanism

By analyzing the Ct-*rpr* interaction in two well-established *in vivo Drosophila* cancer models, we asked whether the combined transcriptional regulation of differentiation and apoptosis repression by Ct could represent a cancer prevention mechanism. In the oncogenic “eyeful” model [16], eye tumors occurred in 72.5% of control flies, with 4.9% of them showing macroscopically visible secondary tumor growths derived from the developing retina (Figure 4H, 4N) due to the eye-specific over-expression of the Notch ligand *Delta* (*Dl*) and the two epigenetic regulators *longitudinals lacking* (*lola*) and *pipsqueak* (*psq*) [16]. In contrast, pre-oncogenic *ey*::*Dl* flies over-expressing *Dl* exclusively in eye tissue [16] never displayed any eye tumors or invasive tumors but only mildly overgrown eyes (Figure 4D, 4N). Eye-specific inhibition of Ct activity alone only caused a small increase in primary and secondary tumor incidences in both sensitized backgrounds (Figure 4E, 4I, 4N), however, these numbers were dramatically increased when Ct function and the ability to activate apoptosis were simultaneously inhibited (Figure 4F, 4G, 4K, 4L, 4M, 4N). Consistently, increased numbers of apoptotic cells were found in tumorous tissue with reduced Ct levels (*ey*::*Dl*;*2xct^RNAi^*) (Figure 4P, 4Q), demonstrating that the coupled regulation of differentiation and apoptosis by a single transcription factor is an important mechanism to suppress cancer.

However, despite increased apoptosis activation in *ey*::*Dl*;*2xct^RNAi^* eye imaginal discs (Figure 4P), which should result in a reduction of tumor growth, tumor formation in these animals was increased (Figure 4N, 4Q). Using the proliferation marker Phosphorylated histone H3 (PH3), we could demonstrate that the tumor growth induced by differentiation loss is due to excessive cell proliferation (Figure 4P), which is in line with previous results [4]. What is the molecular basis for this phenotype? RT-PCR analysis of candidate genes involved in cell cycle and growth control using *ey*::*Dl* and *ey*::*Dl*;*ct^RNAi^* eye imaginal discs revealed a strong induction of phosphoinositide 3-kinase (PI3K) upon Ct depletion (Figure 4O). It has been shown before that PI3K overexpression in the *ey*::*Dl* pre-oncogenic background leads to tumor formation [17] and that PI3K is a limiting factor for *Ras^V12^ Dlg^RNAi^* induced tumor growth [18]. Thus we tested its contribution to tumor formation in Ct-induced oncogenic eyes by reducing its level in *ey*::*Dl*;*2xct^RNAi^* animals. Interestingly, we not only found a rescue of the tumorous eye growth, but also a dramatic increase in the occurrence of smaller eyes in *ey*::*Dl*;*2xct^RNAi^*;*PI3K^RNAi^* animals (Figure 4Q), which is similar to the apoptosis-induced “small eye” phenotype observed upon Ct depletion in the wild-type background (Figure 4B). Taken together, these results show that the Ct-dependent tumor growth is in part mediated by the up-regulation of the PI3K signaling pathway and that this pro-tumorigenic effect counteracts the anti-tumorigenic apoptosis effect of Ct.

### Cell adhesive properties are critical for migratory behavior of tumor cells

**Figure 5:**
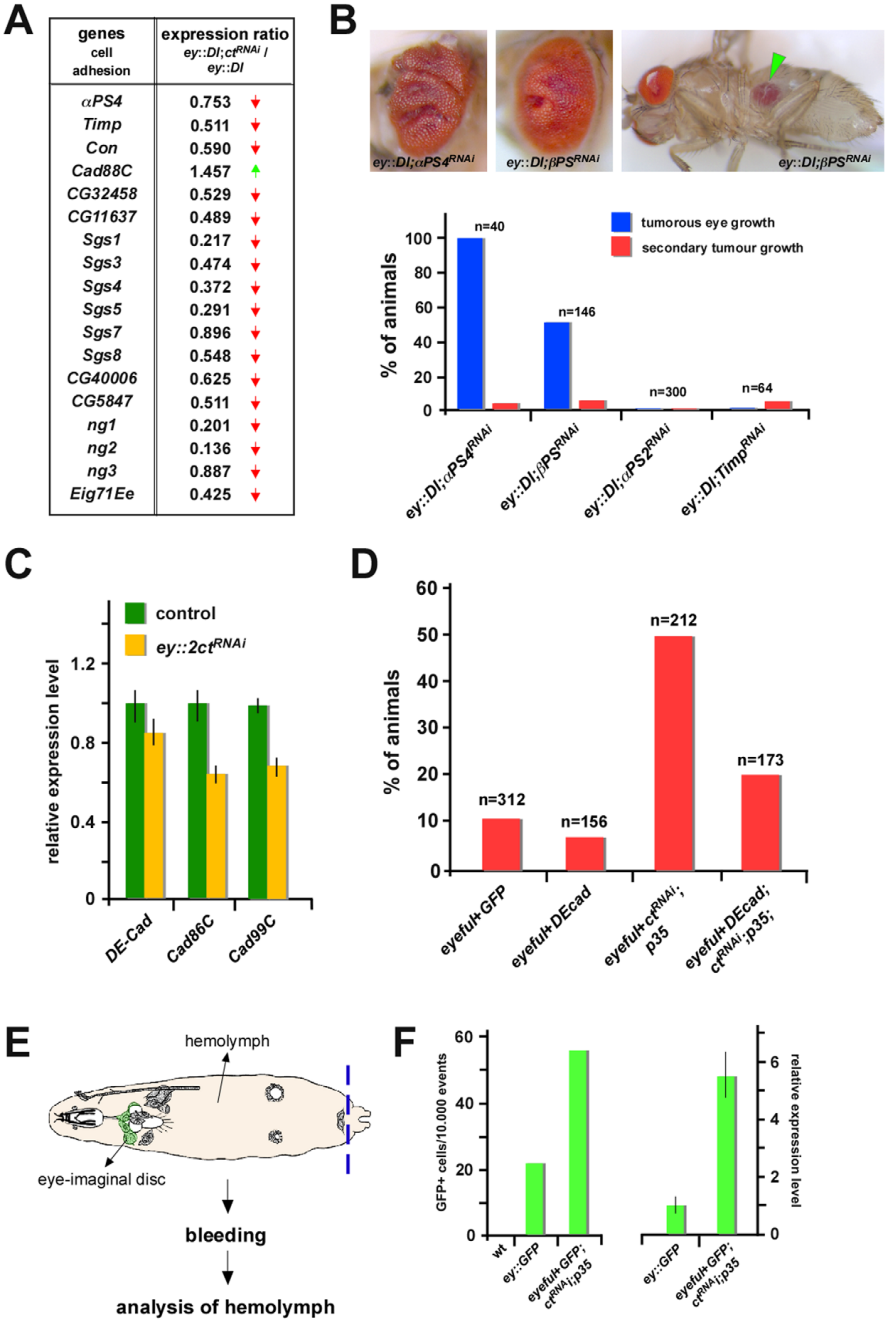
Invasive tumor growth induced by Ct depletion is due to changes in adhesive cell properties. (A) Changes in expression of cell adhesion genes in 3^rd^ instar eye-antennal imaginal discs of *ey*::*Dl*;*2xct^RNAi^* versus *ey*::*Dl* animals identified by expression profiling experiments. Red arrows indicate reduced expression, green arrow induced expression of the respective genes in *ey*::*Dl*;*2xct^RNAi^* animals. (B) Top: Representative pictures of tumor growth in *ey*::*Dl*;*βPSintegrin^RNAi^* and *ey*::*Dl*;*αPS4integrin^RNAi^* flies. Green arrowhead marks secondary tumor growth in the abdomen. Bottom: Quantification of primary and secondary tumor growth in *ey*::*Dl*;*αPS4integrin^RNAi^*, *ey*::*Dl*;*βPSintegrin^RNAi^*, *ey*::*Dl*;*αPS2integrin^RNAi^* and *ey*::*Dl*;*Timp^RNAi^* flies. (C) Relative transcript levels of *DE-Cad*, *Cad86C* and *Cad99C* in eye-antennal discs of 3^rd^ instar larvae of control animals (*Dcr2; ey::lacZ*) and in animals with reduced Ct activity (*Dcr2; ey::2xct^RNAi^*). (D) Quantification of secondary tumor growth rates in different genetic backgrounds. Co-expression of E-Cad strongly reduces invasive tumor growth rates in *eyeful*+*ct^RNAi^;p35* flies. (E) Schematic drawing of a 3^rd^ instar larva expressing GFP in eye-imaginal discs (either *ey*::*GFP* or *eyeful+GFP;ct^RNAi^;p35*). Locations of GFP-labeled eye-imaginal discs and the insect circulatory fluid, the hemolymph, are indicated by arrows. For analysis of the hemolymph, the insect circulatory fluid is extracted by bleeding out the larvae after cutting at the posterior end (indicated by dashed, blue line). (F) Left: Quantification of GFP-positive cells in the hemolymph of wild-type, *ey*::*GFP* and *eyeful*+*GFP*;*ct^RNAi^*;*p35* 3^rd^ instar larvae. Right: Relative *GFP* transcript levels in the hemolymph of *ey*::*GFP* and *eyeful*+*GFP*;*ct^RNAi^*;*p35* 3^rd^ instar larvae.

We found cell clusters expressing the eye differentiation marker ELAV at abnormal, ectopic positions in undifferentiated tissue of 3^rd^ instar eye-antennal discs (Figure S7), and it had been shown before that changes in the adhesive properties of cells are critical in inducing migratory behavior [19], [20]. Consistently, transcriptome profiling experiments revealed a reduction in the expression of cell adhesion genes in eye-imaginal discs of Ct depleted animals exhibiting primary and secondary tumor formation (*ey*::*Dl*;*2xct^RNAi^*) in comparison to control animals (*ey*::*Dl*) (Figure 5A). To test the significance of this finding, we interfered with the function of α-PS4 integrin, one of the genes identified as Ct responsive (Figure 5A), by reducing its expression and the expression of its heterodimeric interaction partner β-PS integrin (*mys*) [21] in the *ey*::*Dl* pre-oncogenic background. We observed an increase in primary and secondary tumor formation in both situations, while reducing the activity of a related but Ct-independent integrin, the α-PS2 integrin (*if*), did not have any effect (Figure 5B). Since decreasing the activity of another Ct responsive cell adhesion gene, namely *Tissue inhibitor of metalloproteases* (*Timp*), also induced an increase in secondary tumors (Figure 5B), we asked if restoration of cell adhesion would be able to rescue this phenotype in the Ct loss-of-function setting. To this end, we expressed one of the major adhesion genes regulated by Ct, *DE-Cad* (Figure 5C), in eye cells of *eyeful*+*ct^RNAi^*;*p35* animals, which display high rates of invasive tumors (Figure 4N, Figure 5D), and observed a reduction of secondary tumor growth rate by more than 50% (Figure 5D). These results demonstrate that regulation of cell adhesiveness is one of the essential Ct-dependent mechanisms to suppress tumor spread. In vertebrates, invasive tumor growth requires the detachment of abnormal cells from tumor tissue and their circulation in the bloodstream [22]. To test if secondary tumor formation mediated by loss of Ct function is dependent on a similar mechanism, we analyzed the hemolymph, the insect “blood”, in our fly lines. Strikingly, we detected a significant increase in GFP-labeled eye-imaginal disc cells in the hemolymph of animals forming invasive tumors (*eyeful*+*GFP*;*ct^RNAi^*;*p35*) in comparison to control animals (*ey*::*GFP*) (Figure 5E, 5F), suggesting that tumor cells in flies indeed circulate through the bloodstream and invade ectopic locations. In sum, these results demonstrate that transcriptional coupling of differentiation and apoptosis is a cell-intrinsic mechanism to ensure normal development and to prevent tumor initiation, progression and invasion, which is at least in part achieved by fine-tuning the adhesive properties of cells required for tissue integrity.

### Antagonistic coupling of cell fate commitment and apoptosis is a general and evolutionary conserved cancer prevention mechanism

**Figure 6:**
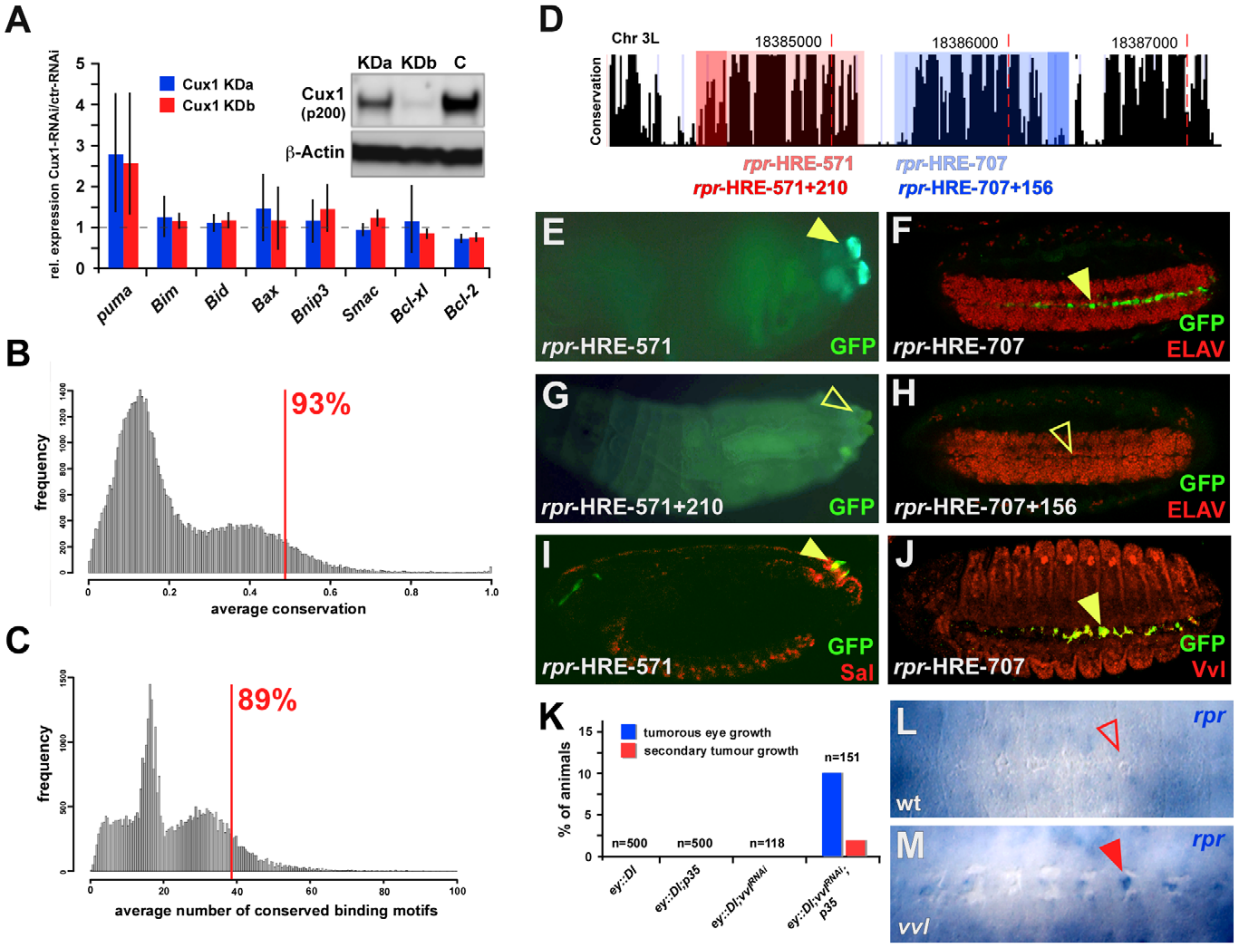
Functional and evolutionary conservation of coupling differentiation and apoptosis on the transcription factor level. (A) Relative mRNA expression of eight apoptosis genes after lenti-virus transduced stable Cux1 (p200) knock-down in human Panc1 cancer cells. RT-PCRs are shown for two independent Cux1 knock-down lines, KDa in blue and KDb in red. Results are shown as the expression ratios between shCux1/shC-treated cells and are representative for three independent experiments. Western blot shows knock-down efficiencies in both independent stable Cux1 (p200) knock-down lines (KDa, KDb) and Cux1 expression in an shRNA control knock-down. Stronger effects of KDa (reduced) versus KDb (almost complete) p200 Cux1 knock-down on target gene expression is very likely due to the processed p110 Cux1 isoform, which can have opposite transcriptional effects to the p200 full-length form [62]. (B, C) Distribution of average conservation (B) and average number of conserved DNA binding motifs per 1000 bp (C) in all non-coding regions of the *D. melanogaster* genome. The red bars highlight the *rpr* intergenic regions, showing that 93% of all *D. melanogaster* non-coding regions are less conserved (B) and 89% of all non-coding regions have fewer conserved DNA binding sites per 1000 bp (C) compared to the *rpr* intergenic regions. (D) Conservation graph of the sequence located downstream of the *rpr* coding region obtained from the UCSC genome browser (http://genome.ucsc.edu/). The following regulatory regions tested are marked in different colors: *rpr*-HRE-571 (light-red), *rpr*-HRE-571+210 (dark-red), *rpr*-HRE-707 (light-blue) and *rpr*-HRE-707+156 (dark-blue). (E–H) Reporter gene expression driven by the fragments described above. The *rpr*-HRE-571 enhancer drives reporter gene expression in the PS (E), which is abolished in the *rpr*-HRE-571+210 reporter line (G). Similarly, reporter gene expression in CNS midline cells in the *rpr*-HRE-707 line (F) is completely suppressed in the *rpr*-HRE-707+156 transgenic line (H). Closed, yellow arrowheads in (E) and (F) mark presence of reporter gene expression, whereas open, yellow arrowheads in (G) and (H) mark absence of GFP expression. (I, J) Co-localization of GFP with Sal in the *rpr*-HRE-571 (I) and with Vvl in the *rpr*-HRE-707 (J) reporter lines. (K) Quantification of primary and secondary tumor formation in different genetic backgrounds. Only when Vvl function is reduced and apoptosis is simultaneously inhibited, tumors and metastasis develop. (L, M) *rpr* transcripts are not found in CNS midline cells of stage 14 wild-type embryos (L) but in *vvl* mutants (M).

We next explored whether the effective regulation of programmed cell death by Ct has been conserved during evolution. The vertebrate homologue of Cut, Cux1, has a well-documented function in cell differentiation during normal development as well as in tumor initiation and progression in specific cancer types [23]. In addition, several studies show that Cux1 represses apoptosis during normal vertebrate development [24], [25], [26], and just recently it has been demonstrated that Cux1 knock-down leads to activated apoptosis and to reduced growth of xenograft tumors *in vivo* [25], [27]. To further investigate the mechanistic basis of Cux1 function in mediating apoptosis repression in vertebrates, we suppressed Cux1 in Panc1 pancreatic cancer cells (Figure 6A) and determined the transcriptional response of human apoptosis genes. Strikingly, mRNA levels of the pro-apoptotic gene *puma* were consistently elevated, whereas the anti-apoptotic gene *Bcl-2* was down-regulated upon Cux1 depletion (Figure 6A). Since BH3-only proteins, like Puma, and Bcl-2 are important for the release of the vertebrate functional equivalent of Rpr, Smac/DIABLO, from mitochondria [28] and since Cux1 binding sites are present close to the *puma* coding region (Table S1), these results suggest that the regulatory wiring of differentiation and apoptosis, at the level of Cut, is functionally conserved in vertebrates.

**Figure 7:**
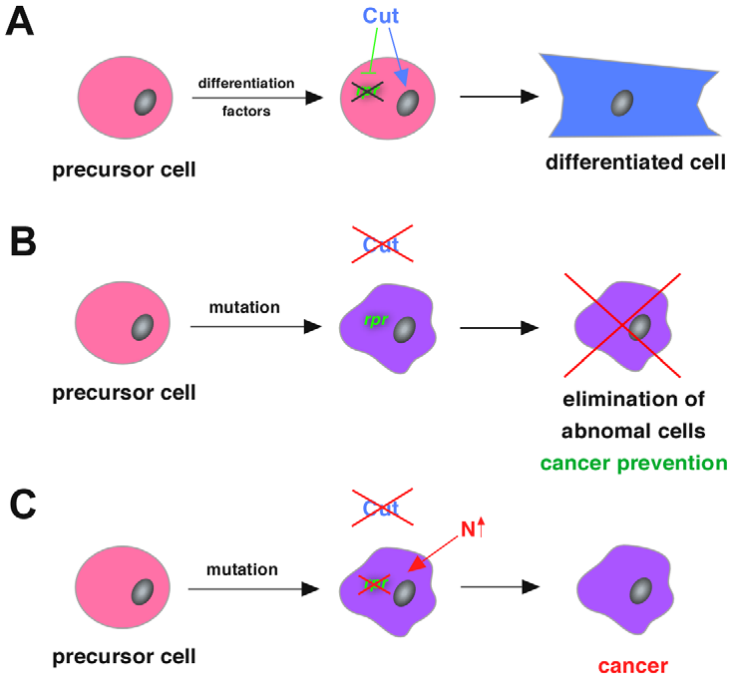
Model of cancer prevention mechanism by cell fate specifying transcription factors like Cut. (A) During normal development, cell-type specification factors like Cut ensure the survival of cells by repressing apoptosis while at the same time these factors also induce a specific differentiation program, which generates cells with a specific terminal cell fate. (B) In the case of a mutation in a cell-type specification factor those cells unable to differentiate, which are potentially harmful to the organism, are removed by releasing apoptosis repression conferred by the same cell-type specification factor. Thus, the transcriptional coupling of differentiation and apoptosis regulation represents a very fast and efficient cancer prevention mechanism. (C) Together with other mutations creating a sensitized background, like the over-activation of the Notch (N) signaling pathway, cells that acquire the inability to differentiate and a resistance to apoptosis activation, two important hallmarks of cancer [1], [2], very easily develop into cancer cells.

Does this regulatory layout represent a general mechanism employed by other differentiation factors? This would require a whole suite of cell-type specifying transcription factors to repress cell death genes by interacting with distinct enhancer modules located in their regulatory regions. In addition, these modules should follow a similar functional logic to the *rpr*-HRE-571 enhancer, in that cell-type specific gene activation is counteracted by strong repressing inputs from linked cis-elements (Figure 1L–1O). In line with this, we found that a different conserved enhancer module on the *Drosophila rpr* regulatory region (*rpr*-HRE-707) drove expression in CNS midline cells of stage 14 embryos (Figure 6F), which never express *rpr* at this and subsequent developmental stages (Figure 6L) [29]. However, extending the enhancer to include additional cis-elements (*rpr*-HRE-707+156) (Figure 6D) resulted in loss of enhancer activity (Figure 6H). Using the JASPAR database [30], we found consensus binding sequences for POU-domain containing transcription factors on the extended enhancer module, and one of these factors, Ventral veins lacking (Vvl), is known to function in midline glial cells and to repress apoptosis [31], [32]. Our analysis revealed a partial overlap of Vvl and reporter gene expression in *rpr*-HRE-707 embryos (Figure 6J), and consistently ectopic *rpr* transcripts were detected in several midline cells of *vvl* mutants (Figure 6L, 6M). Due to the existence of GFP-positive cells not expressing Vvl (Figure 6J), we assume that not only Vvl but also other POU transcription factors interact with the *rpr*-HRE-707+156 enhancer to repress *rpr* transcription in midline cells. Revisiting the *rpr*-HRE-571 enhancer module revealed that extension of the enhancer also led to a complete loss of reporter activity (compare Figure 1L, Figure 6E, 6G). Thus, complete repression of *rpr* transcription in the PS requires two inhibitory inputs: one active in filzkörper cells, which we had identified to be mediated by Ct, and one so-far unknown repressor functional in stigmatophore cells. Importantly, the functional analogy of Vvl and Ct also extended to the tumor suppression activity, since, like in the case of Ct (Figure 4N), primary and secondary tumor frequencies were increased when the ability to activate apoptosis and Vvl function was impaired at the same time (Figure 6K). Furthermore, we identified two unrelated cell-type specifying transcription factors in addition to Ct and Vvl, which showed similar behavior with regards to tumor suppression (Figure S8). Together with the fact that the regulatory sequences flanking the *Drosophila rpr* coding region show significantly less sequence divergence than expected and a high occurrence of conserved transcription factor binding motifs (Figure 6B, 6C), these findings lead us to propose that coupling of differentiation and cell death repression via a single transcription factor represents a general cancer prevention mechanism (Figure 7), which could be employed by a large number of developmental regulators in diverse organisms.

## Discussion

Programmed cell death is an integral aspect of animal development [33]. Genetic studies in *C. elegans*, *Drosophila* and mouse have shown that apoptosis is used to sculpt tissues and to remove excessive and unwanted cells, thus defining the morphology required for diverse physiological functions [34]. In this context, apoptosis is usually regulated by cell signaling pathways [33], [35], [36]. In addition to its role in tissue morphogenesis, apoptosis is also required to eliminate potentially deleterious cells, which in most cases involves complex multi-step control mechanisms [33], [37]. One such situation generating harmful cells is the inability to differentiate or adopt the appropriate cell fate, which very often results in uncontrolled cell proliferation and cancer development, and thus requires the immediate killing of these cells. However, even though it is established that apoptosis is a protective mechanism against tumorigenesis in cases of aberrant cell differentiation [1], [34], [38], the interplay of the two processes at the mechanistic level has remained unclear. In our study, we show that the simultaneous and antagonistic regulation of differentiation and apoptosis is a hard-wired developmental program and carried out by individual transcription factors, such as Cut. Our results demonstrate that impairment of differentiation in the cell lineage specified by Cut instantaneously triggers locally restricted apoptosis by releasing transcriptional repression of the pro-apoptotic gene *rpr* in these cells. Due to its immediate effect, the coupling of differentiation and apoptosis on the transcriptional level represents one of the fastest and most direct mechanisms to eliminate abnormal cells *in status nascendi* and thereby immediately interferes with their potential to develop into harmful cells.

Interestingly, apoptosis induction as a consequence of aberrant cell-type specification is not only mediated by the cell death promoting gene *rpr* but also by *hid* [8]. However, despite the same trigger, which is the inability to properly differentiate, the transcriptional basis for inducing the expression of one of these two apoptosis genes seems to be quite different: in *Drosophila* early developmental mutants only the expression of the pro-apoptotic gene *hid* is up-regulated [8], whereas our study shows that exclusively the transcription of *rpr* is induced when a factor specifying a distinct cell type is lost. Although it is currently unknown how *hid* expression is regulated at the transcriptional level, this raises the possibility that the apoptosis gene *hid* acts a safeguard when broad positional information at the onset of embryogenesis is absent, whereas *rpr* might take over this function later in development when individual and specific cell types are defined by transcription factors restricting cell fate choices.

Given the well-known role of the vertebrate homologue of Cut, Cux1, in tumor initiation and progression in specific cancer types [23], we addressed whether the switch function of the cell specification factor Cut is also relevant in a pathological context. We found that simultaneous inhibition of Cut function and apoptosis within a sensitized background increases tumor formation and metastasis to secondary sites in the animal. In contrast, down-regulation of Cut and inhibition of apoptosis in a normal developmental context, such as in the *Drosophila* PS or the developing eye, only results in the survival of the Cut deprived cells, but not in tumor development. These results demonstrate that cells, which are unable to undergo the cell lineage-specific differentiation program, have to be eliminated, since they have the potential to develop into cancerous cells when other genetic or micro-environmental changes accumulate [19], [27], [39]. But why do differentiation-deprived cells form tumors in a cancer-prone tissue environment despite the ability to activate the apoptotic rescue pathway? This is due to the fact that the transcription factor Cut, as part of its selector gene function, coordinately regulates multiple cellular processes, including differentiation, apoptosis, cell adhesion, but also proliferation, which are all required for proper cell fate specification and the maintenance of a differentiated state (thereby preventing tumor formation). If, however, Cut activity is abolished, all its downstream functions are affected, leading not only to the activation of apoptosis, but also to reduced differentiation and adhesion properties and the activation of cell proliferation, which is, in the case of Cut, mediated (at least in part) by the PI3K signaling pathway. Thus, loss of Cut function stimulates tumor growth in a sensitized background, since the pro-tumorigenic effects of deregulated proliferation and cell adhesiveness out-compete the anti-tumorigenic apoptosis effects at work. However, when the anti-tumorigenic effect is eliminated in the differentiation-compromised cancer tissue, tumorigenesis is strongly enhanced, which resembles a prevalent situation in aggressive human cancers characterized by the loss of differentiation, the resistance to apoptosis activation and the mis-regulation of adhesion properties [1], [40], [41].

Several lines of evidence suggest that the dual role of Cut in differentiation and apoptosis for cancer prevention is conserved in evolution. First of all, the two vertebrate homologues of Cut, Cux1 and Cux2, code for homeobox-containing transcription factors, which are crucially involved in cell-type specific terminal differentiation [14], [23], [42]. Both, Cux1 and Cux2, have similar binding specificities to *Drosophila* Cut [43], they also operate as transcriptional repressors and activators of genes in multi-lineage differentiation pathways [26] and, like *Drosophila* Cut, they act as downstream effectors of the Notch signaling pathway [44], [45]. In addition to their well-established role in development and differentiation, there are also several examples linking the vertebrate Cut homologue Cux1 to apoptosis and cancer. First, inhibition or partial disruption of Cux1 function in mice leads to increased apoptosis rates *in vivo* [24], [26]. Second, Cux1 regulates normal hematopoiesis, in part by modulating the levels of survival and apoptosis factors [26]. Third, Cux1 plays a prominent role in cancer progression [23]. And fourth, induced down-regulation of Cux1 in subcutaneous xenograft tumors leads to activation of apoptosis and to reduced tumor growth [25]. Our results now show that the Cut-Rpr regulatory wiring of apoptosis and differentiation is conserved in vertebrates. In mammalian cells, the Rpr functional homologue, Smac/DIABLO, which is normally compartmentalized within mitochondria, has to be released to execute its pro-apoptotic function by binding to and inactivating Inhibitors of Apoptosis (IAPs) [46]. This process requires the permeabilization of the outer mitochondrial membrane (MOMP), which is achieved by the interaction of pro-apoptotic proteins like Puma with anti-apoptotic proteins like Bcl-2, which normally inhibit MOMP [47]. We now show that down-regulation of Cux1 in pancreatic cancer cell lines leads specifically to the transcriptional induction of the pro-apoptotic gene *puma* and the down-regulation of the anti-apoptotic gene *Bcl-2*. Thus, two crucial regulators for Smac/DIABLO release are controlled by Cux1 on the transcriptional level, showing that the basic design principle of the Cut-Rpr regulatory wiring is conserved but has been adapted to the system requirements in evolution. In future, it will be intriguing to study this mechanism in diverse cellular backgrounds, including stem cells, which neither die nor differentiate.

## Materials and Methods

### Bioinformatics

To identify Abd-B binding sites we used the method of Wasserman and Sandelin (2004) [48] with the Abd-B Position Frequency Matrix [49] (http://jaspar.genereg.net/) and 90% cut-off. Abd-B binding site clusters were identified if at least three Abd-B sites were present in a 400 bp window. Conserved enhancers were identified using PhastCon score [50]. Within conserved regions, Ct binding sites were identified using published sequence data [49]. Vvl and Cux1 binding sites were identified using the Vvl and Cux1 Position Frequency Matrices available at the JASPAR database [49] (http://jaspar.genereg.net/).

### Genetics

*Drosophila melanogaster* strain *Oregon R* was used as wild type. Amorphic allele *ct^db7^/FM7* [51], *ems-Gal4* and *ems-Gal4, UAS-GFP/TM6B* [13] were obtained from J. Castelli-Gair Hombria, *UAS-Dcr2; ey-Gal4* from B. Dickson, *eq-Gal4/TM6B* [52] from H. Pi, *UAS-Apoliner5* [11] from J. P. Vincent, *UAS-ct^EHK2^/CyO* [53], *UAS-ct^RNAi^; UAS-ct^RNAi^* (Grueber and Jan, unpublished) from Y.N. Jan and *ey-Gal4, UAS-Dl/CyO* and *eyeful* flies (*ey-Gal4, GS88A8, UAS-Dl/CyO*) from M. Domiguez [16]. *UAS-Dcr2; C96-Gal4* (BL-25757), *UAS-CD8::GFP* (BL-5130) from Bloomington stock center. *GMR-Gal4*, *UAS-p35*, *UAS-Abd-B*, *arm-Gal4*, *UAS-rpr*, *UAS-lacZ* were described elsewhere [54], [55], [56], [57]. Other UAS-RNAi lines were obtained either from BDSC, VDRC or TRiP: *DE-Cad* (v8024), *rpr* (v12045), *vvl* (JF02126), *gro* (v6316), *H* (v24466), *αPS2* (*if*) (BL27544), *αPS4* (v109783), *βPS* (*mys*) (HMS00043), *PI3K* (v107390) and *Timp* (v109427). Five independent transgenic lines were analyzed for each reporter construct.

### Mammalian cell culture and lentivirus-mediated Cux1 knock-down

Panc-1 human pancreatic cancer cells (Department of Surgery, Medical Faculty, University of Heidelberg) and 293 T cells were maintained in DMEM supplemented with 10% fetal bovine serum, 2 mM L-glutamine, non-essential amino acids, 100 U/ml penicillin, 100 U/ml streptomycin, and 0.25 µg/ml amphotericin B. shRNA directed against human Cux1 was generated using the following complementary oligonucleotides (forward and reverse):

Cux1_KDa:

5′CCGGAAGAAGAACACTCCAGAGGATCTCGAGATCCTCTGGAGTGTTCTTCTTTTTTTG3′ and

5′AATTCAAAAAAAGAAGAACACTCCAGAGGATCTCGAGATCCTCTGGAGTGTTCTTCTT3′;

Cux1_KDb:

5′CCGGAAGAATCTTCTCGTTTGAAACCTCGAGGTTTCAAACGAGAAGATTCTTTTTTTG3′ and

5′AATTCAAAAAAAGAATCTTCTCGTTTGAAACCTCGAGGTTTCAAACGAGAAGATTCTT3′;

shRNA control (C),

5′CCGGAATTGCCAGCTGGTTCCATCACTCGAGTGATGGAACCAGCTGGCAATTTTTTTG3′ and

5′CCGGAATTGCCAGCTGGTTCCATCACTCGAGTGATGGAACCAGCTGGCAATTTTTTTG3′.

pLKO lentiviral vectors containing shRNA were transfected into 293 T cells together with psPAX2 (packaging vector) and pMD2.G (VSV-G envelope protein expression vector) using the calcium-phosphate transfection kit (Sigma). Panc-1 cells were infected using lentivirus-containing 293 T cell supernatant and Cux1 protein levels were assessed by Western blotting using anti-Cux1 (Sigma-Aldrich) and anti-β-Actin (GeneTex) antibodies.

### Plasmid constructs

All enhancer fragments were PCR amplified from genomic DNA and cloned in *pHPelican-GFP* [58] or *pHPelican-GFP_DEST* [59]. For binding site mutations, a two-step overlap PCR was performed. For mutating Ct binding sites within the *rpr*-HRE-571-S2 enhancer fragment, mutation introduced into the Cut consensus sequences were identical to the ones introduced into the EMSA probes (see below). Primer sequences are available upon request.

### Real-time PCR

Real Time PCR was performed following standard protocols using SYBR green. Expression was normalized to GAPDH for mammalian cells and to endogenous *actin5C* mRNA for imaginal disc analysis. Relative expression levels are based on three biological replicates.

### Histology and scanning electron microscopy

*Drosophila* embryos were fixed as described [56]. Eye discs or wing discs were dissected in PBS and fixed in 4% paraformaldehyde/PBS for 10 min for immunostaining. *In situ* hybridization and immunochemistry were performed as described [56]. Fluorescent mRNA/protein double labeling and fluorescent duplex *in situ* hybridizations were done as described previously [60]. Primary Antibodies used were: mouse anti-Ct 2B10 (1∶200, DSHB), mouse anti-Crb cq4 (1∶200, DSHB), rat anti-DE-Cad DCAD2 (1∶100, DSHB), mouse anti-ELAV (1∶200, DSHB), rat anti-Sal (1∶800 kind gift from R. Barrio), rabbit anti-PH3 (1∶200, Santa Cruz), rabbit anti-Cleaved Caspase-3 (1∶50 Cell Signalling), mouse anti-GFP (1∶1000, Roche), rabbit anti-GFP (1∶2000, Sigma), anti-DIG POD (1∶200, Roche), Streptavidin HRP (1∶200, PerkinElmer). Scanning electron microscopy, Acridine Orange staining and cuticle preparation were carried out as described in Lohmann et al. (2002) [55] and Zhai et al. (2010) [61]. TUNEL assay was performed with the In Situ Cell Death Detection Kit (TMR) from Roche according to the manufacturer's instruction.

### Protein purfication and electrophoretic mobility shift assays (EMSA)

Cut CR3HD (4849–5412 of ctRA from Y.N. Jan) was cloned into *pMAL2-c2x* vector (NEB) and expressed as Maltose Binding Protein (MBP) fusion proteins. EMSAs were carried out as described in Stöbe et al. (2009) [56]. The following oligonucleotides (S2 subfragment) were used for analyzing the Cut binding sequence in EMSA (only forward strand is shown):

**Wild-type.** 5′GCACTTTTGCCTGCAGT**TC**AACTCGGT**TC**AGT**TC**GGTTGTGTCATAAAAAATC3′

**Mutated.** 5′GCACTTTTGCCTGCAGT**GG**AACTCGGT**GG**AGT**GG**GGTTGTGTCATAAAAAATC3′

Cut consensus sequences are underlined, exchanged nucleotides in the mutated versus the wild-type sequence are shown in bold.

### Quantification of GFP labeled eye cells in hemolymph of 3^rd^ instar larvae

*ey::CD8-GFP* or eyeful+*CD8-GFP*;*ct^RNAi^*;*p35* 3^rd^ instar larvae were dissected by rupturing the larval cuticle at the posterior end with a pair of fine forceps, the hemolymph was collected in ice-cold Schneider's medium (Invitrogen GIBCO) containing 1× Complete protease inhibitor mixture (Roche). Hemolymph cells were analyzed via FACSAria to quantify GFP-labeled cells circulating within the hemolymph. In addition, *GFP* mRNA levels within the hemolymph were measured by qRT-PCR.

### Microarray

Eye-antennal discs of *ey::Dl* or *ey::Dl::2xct^RNAi^* 3^rd^ instar larvae were dissected in cold PBS, total RNA was extracted using standard procedures. Microarray analysis was conducted at the Genomics Core Facility, EMBL, Heidelberg, Germany. Microarray data analysis was performed using the R package as described previously [54].

## Supporting Information

Figure S1 Cut directly represses *rpr* transcription in a cell-autonomous manner. (A–D) Expression of the posterior spiracle markers Spalt (Sal) (green), which labels the stigmatophore precursor cells, and Cut (Ct) (red), which marks the spiracular chamber and filzkörper precursor cells, in stage 11 (A), stage 13 (B), stage 14 (C) and stage 16 (D) embryos. In (A), (B) and (D) lateral views of the posterior spiracle primordia are shown, whereas in (C) a dorsal view is presented. (E, F) *rpr* mRNA expression (green) in posterior spiracle primordia of stage 14 wild-type (E) and *ct* mutant (F) embryos is shown (lateral view). Spalt (Sal) protein (blue) labels stigmatophore precursor cells, Cut (Ct) protein (red, nuclear) marks spiracular chamber and filzkörper precursor cells and the apical membrane marker Crb (red) outlines the cells. Small, white arrow in (F) marks additional tracheal cells found at the posterior end in *ct* mutant embryos; yellow circle in (F) highlights *rpr* expression in *ct* mutant embryos. (G) EMSA using S2 sub-fragment with Ct binding sites either in wild-type (wt probe) or mutated (mut. probe) version and no protein (−), purified MBP protein (M), and purified Cut-MBP fusion protein consisting of the Cut repeat 3 and the Cut homeodomain (C). The black arrowheads indicate the specific DNA-protein complexes, the black arrow highlights unspecific DNA-protein complex. Loading of equal amounts of labeled wild-type and mutated oligonucleotides is illustrated by formation of comparable amounts of unspecific DNA-protein complex (indicated by black arrow).
(JPG)

Figure S2 Ct represses *rpr* transcription and apoptosis activation. (A–F) *rpr* RNA expression in stage 11 wild-type (A, C, E), *ct^db7^* (B, D) and *arm::ct* (F) embryos. *rpr* transcription is ectopically activated in the posterior spiracle primordium (B) and the gut primordium (D) in *ct* mutant embryos (marked by red arrowheads), and is globally repressed when Ct is ubiquitously mis-expressed (F). (G, H) Acridine Orange (AO) staining of stage 13 wild-type (G) and *ct* mutant (H) embryos highlights up-regulation of programmed cell death in the PS primordium of *ct* mutant embryos. (I–N) *hid* (I, J), *grim* (K, L), *skl* (M, N) RNA expression in stage 11 wild-type (I, K, M) and *ct^db7^* (J, L, N) mutant embryos. Red boxes indicate posterior spiracle primordium in respective embryos.
(JPG)

Figure S3 Cut directly represses *rpr* and apoptosis in the PS primordium. (A–D) GFP expression in the posterior spiracle primordium of different reporter lines at developmental stage 15. Spalt (Sal) and Cut (Ct) proteins label stigmatophore (blue) or spiracular chamber and filzkörper precursor cells (red). Closed, yellow arrowheads in (C) and (D) mark reporter gene expression in filzkörper cells, whereas open, yellow arrowheads in (A) and (B) mark missing GFP expression. (E–H) Single color images of the different reporter lines showing only Sal expression. (I–L) Single color images of the different reporter lines showing only Ct expression. (M–P) Single color images of the different reporter lines showing only GFP expression.
(JPG)

Figure S4 Location and conservation of the *rpr*-HRE-571 element. (A) Conservation graph of the *rpr* intergenic region obtained from the UCSC genome browser (http://genome.ucsc.edu/). The *rpr*-HRE-571 element, which is marked by a light-red box (3 L: 18384438..18385008), is located 6 kb downstream of the *rpr* coding sequence (marked by a dark-red box). The coding region of the pro-apoptotic gene *grim* is marked by a dark-blue box. (B) Alignment of the *rpr*-HRE-571 region from five different *Drosophila* species (*D. melanogaster*, *D. yakuba*, *D. pseudoobscura*, *D. virilis*, *D. grimshawi*). Abd-B binding sites are marked by purple, Ct binding sites by orange boxes.
(JPG)

Figure S5 Ct function is required for filzkörper differentiation. The following genotypes are shown: wild type (A, D and G), *ct^db7^; ems::GFP* (B, E and H) and *ct^db7^; ems::p35;GFP* (C, F and I). (A–C) Cuticle preparations of the different genotypes with focus on the posterior spiracle of 1^st^ instar *Drosophila* larvae. Closed, orange arrowhead in (A) marks the filzkörper, whereas open, orange arrowheads in (B) and (C) indicate the absence of this structure in the respective genotypes. (D–F) Ct and Crb stainings in the respective embryos are shown to highlight the morphology of the filzkörper in the different genotypes. (G–I) DE-Cad staining in the respective genotypes. Closed, red arrowheads in (G) indicate the presence of the filzkörper, whereas open, red arrowheads in (H) and (I) highlight the absence of this structure in *ct^db7^; ems::GFP* (H) and *ct^db7^; ems::p35;GFP* (I) embryos.
(JPG)

Figure S6 Ct represses apoptosis in wing margin bristles and in external sensory organs of the notum. (A–D) Close-up of *Drosophila* notum in wild-type (A), *Eq*::*ct^RNAi^* (B), *Eq*::*ct^RNAi^*; *p35* (C) and in *Eq*::*ct^RNAi^*; *rpr^RNAi^* animals (D). Open, yellow circles in (B, C and D) mark the absence of external sensory organs, whereas closed, yellow circle in (A) highlights the presence of these structures in the different genotypes. Note that in (B) most bristles are missing, whereas in (C) and (D) some bristles form, which have cell polarity defects. Expression of Cut in mechanosensory organs of the notum has been shown before [63]. (E–H) Close-up of *Drosophila* adult wing with focus on wing margin between the wing veins L2 and L3 in wild-type (E), *C96*::*ct^RNAi^* (F), *C96*::*ct^RNAi^*; *p35* (G) and in *C96*::*ct^RNAi^*; *rpr^RNAi^* (H) animals. Closed, blue arrowheads in (E), (G) and (H) highlight the presence of mechanosensory bristles at the wing margin, whereas the open, blue arrowhead in (F) marks their absence in the respective genotype. Importance of Cut function for wing margin development has been shown before [64], [65], [66], [67]. Despite the fact that Cut is expressed in a narrow region along the wing margin [64], [65], [66], [67], we observed a loss of cells outside that region. One likely explanation for this phenotype is the known requirement of Cut to maintain expression of the secreted factor Wingless (Wg) at the wing margin [64], [65], [66], [67], thus we assume that neighboring cells which normally receive the Wg signal undergo apoptosis in a cell non-autonomous manner. (I, J) Quantification of mechanosensory bristles on notum (I) and between wing veins L2 and L3 (J) in the different genetic backgrounds. 15–20 flies were scored for each genotype.
(JPG)

Figure S7 Invasiveness of Ct depleted cells. (A–D) Co-localization of the eye differentiation marker Embryonic Lethal Abnormal Vision (ELAV) and the proliferation marker Phosphorylated histone H3 (PH3) in 3rd instar eye-antennal discs. Blue circle in (D) marks loss of ELAV expression in *eyeful*::*ct^RNAi^* 3^rd^ instar eye-antennal discs, yellow circle in (B) marks ELAV-positive cells at ectopic location in *eyeful*::*ct^RNAi^;p35* 3^rd^ instar eye-antennal discs.
(JPG)

Figure S8 Simultaneous regulation of differentiation and apoptosis represents a general cancer prevention mechanism. Top: Representative pictures of tumorous eye growth in flies of indicated genotypes. Bottom: Quantification of primary tumor growth in the respective genotypes. Genes tested were selected based on their function as cell-type specifying transcriptional regulators active in the *Drosophila* eye. Genes: *vvl*: *ventral veins lacking*; *gro*: *groucho*; *ct*: *cut*; *H*: *Hairless*.
(JPG)

Table S1 Putative binding sites for vertebrate Cux1 within the non-coding regions of the *puma* gene.
(DOC)
