# Supplementary figures and images for: Antagonistic Regulation of Apoptosis and Differentiation by the Cut Transcription Factor Represents a Tumor-Suppressing Mechanism in Drosophila
Source: PLoS Genet. 2012 Mar 15;8(3):e1002582. doi: 10.1371/journal.pgen.1002582 (PMC3305397; doi:10.1371/journal.pgen.1002582)

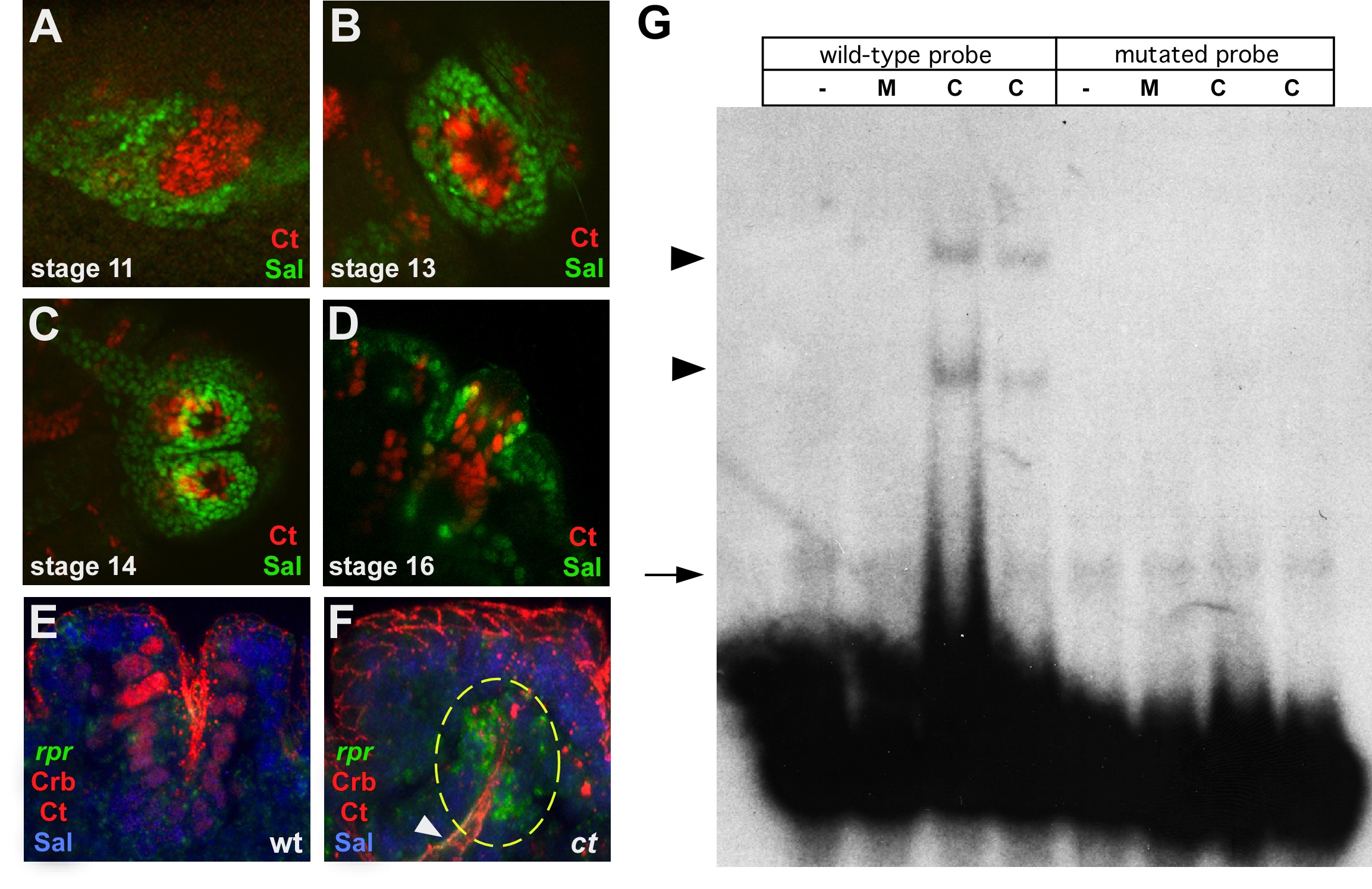

Supplement: Figure S1 — Cut directly represses rpr transcription in a cell-autonomous manner. (A–D) Expression of the posterior spiracle markers Spalt (Sal) (green), which labels the stigmatophore precursor cells, and Cut (Ct) (red), which marks the spiracular chamber and filzkörper precursor cells, in stage 11 (A), stage 13 (B), stage 14 (C) and stage 16 (D) embryos. In (A), (B) and (D) lateral views of the posterior spiracle primordia are shown, whereas in (C) a dorsal view is presented. (E, F) rpr mRNA expression (green) in posterior spiracle primordia of stage 14 wild-type (E) and ct mutant (F) embryos is shown (lateral view). Spalt (Sal) protein (blue) labels stigmatophore precursor cells, Cut (Ct) protein (red, nuclear) marks spiracular chamber and filzkörper precursor cells and the apical membrane marker Crb (red) outlines the cells. Small, white arrow in (F) marks additional tracheal cells found at the posterior end in ct mutant embryos; yellow circle in (F) highlights rpr expression in ct mutant embryos. (G) EMSA using S2 sub-fragment with Ct binding sites either in wild-type (wt probe) or mutated (mut. probe) version and no protein (−), purified MBP protein (M), and purified Cut-MBP fusion protein consisting of the Cut repeat 3 and the Cut homeodomain (C). The black arrowheads indicate the specific DNA-protein complexes, the black arrow highlights unspecific DNA-protein complex. Loading of equal amounts of labeled wild-type and mutated oligonucleotides is illustrated by formation of comparable amounts of unspecific DNA-protein complex (indicated by black arrow). (JPG) [file pgen.1002582.s001.jpg]

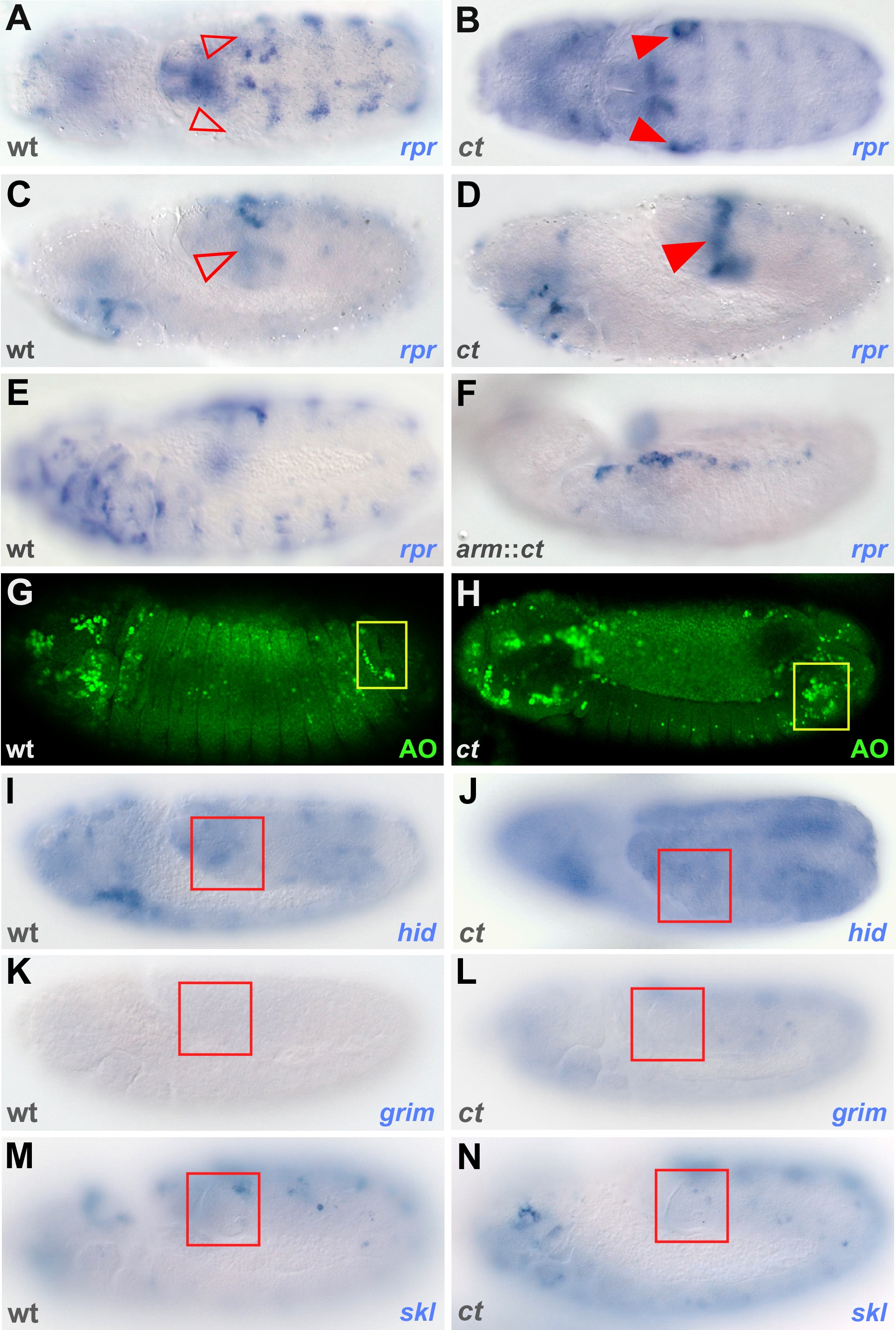

Supplement: Figure S2 — Ct represses rpr transcription and apoptosis activation. (A–F) rpr RNA expression in stage 11 wild-type (A, C, E), ctdb7 (B, D) and arm::ct (F) embryos. rpr transcription is ectopically activated in the posterior spiracle primordium (B) and the gut primordium (D) in ct mutant embryos (marked by red arrowheads), and is globally repressed when Ct is ubiquitously mis-expressed (F). (G, H) Acridine Orange (AO) staining of stage 13 wild-type (G) and ct mutant (H) embryos highlights up-regulation of programmed cell death in the PS primordium of ct mutant embryos. (I–N) hid (I, J), grim (K, L), skl (M, N) RNA expression in stage 11 wild-type (I, K, M) and ctdb7 (J, L, N) mutant embryos. Red boxes indicate posterior spiracle primordium in respective embryos. (JPG) [file pgen.1002582.s002.jpg]

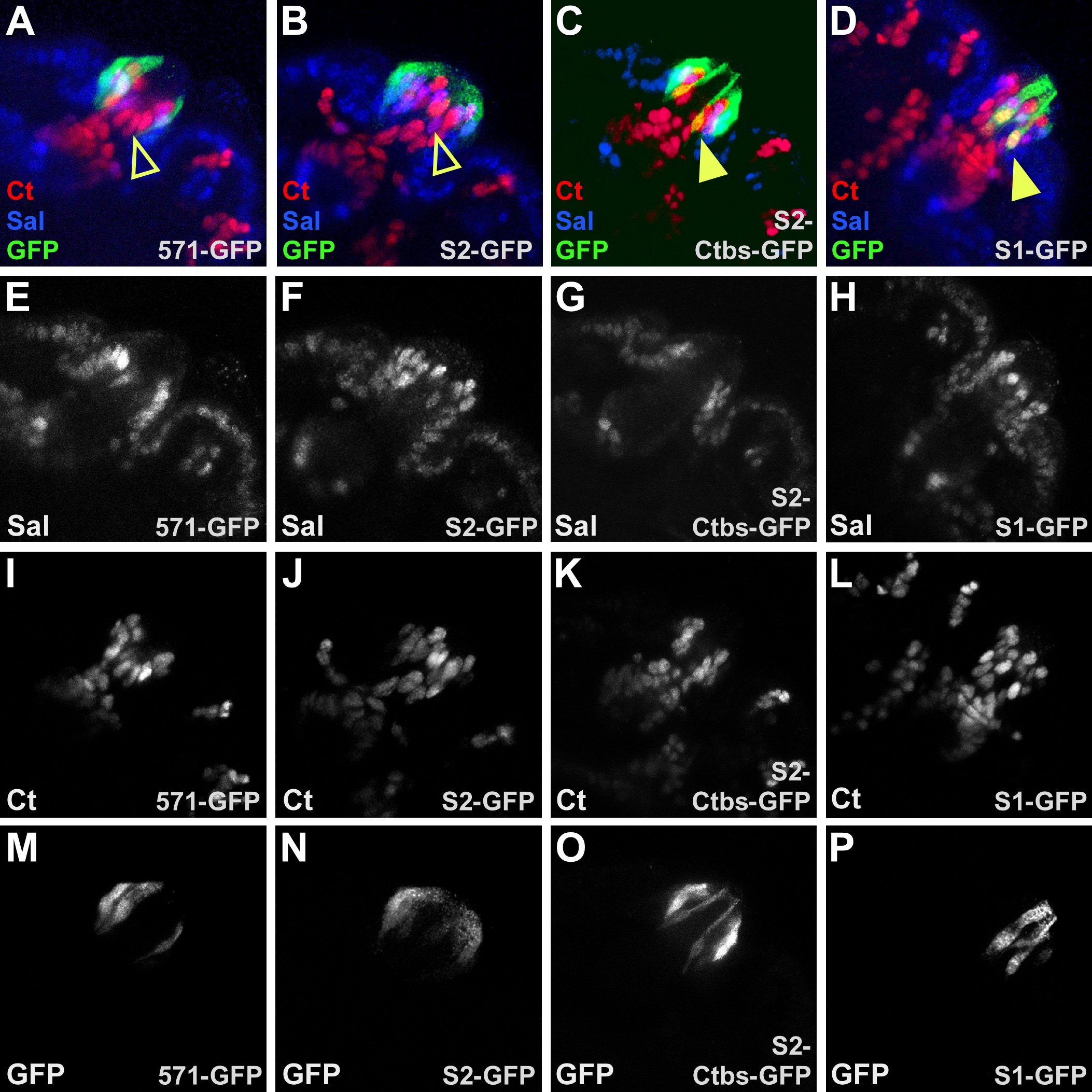

Supplement: Figure S3 — Cut directly represses rpr and apoptosis in the PS primordium. (A–D) GFP expression in the posterior spiracle primordium of different reporter lines at developmental stage 15. Spalt (Sal) and Cut (Ct) proteins label stigmatophore (blue) or spiracular chamber and filzkörper precursor cells (red). Closed, yellow arrowheads in (C) and (D) mark reporter gene expression in filzkörper cells, whereas open, yellow arrowheads in (A) and (B) mark missing GFP expression. (E–H) Single color images of the different reporter lines showing only Sal expression. (I–L) Single color images of the different reporter lines showing only Ct expression. (M–P) Single color images of the different reporter lines showing only GFP expression. (JPG) [file pgen.1002582.s003.jpg]

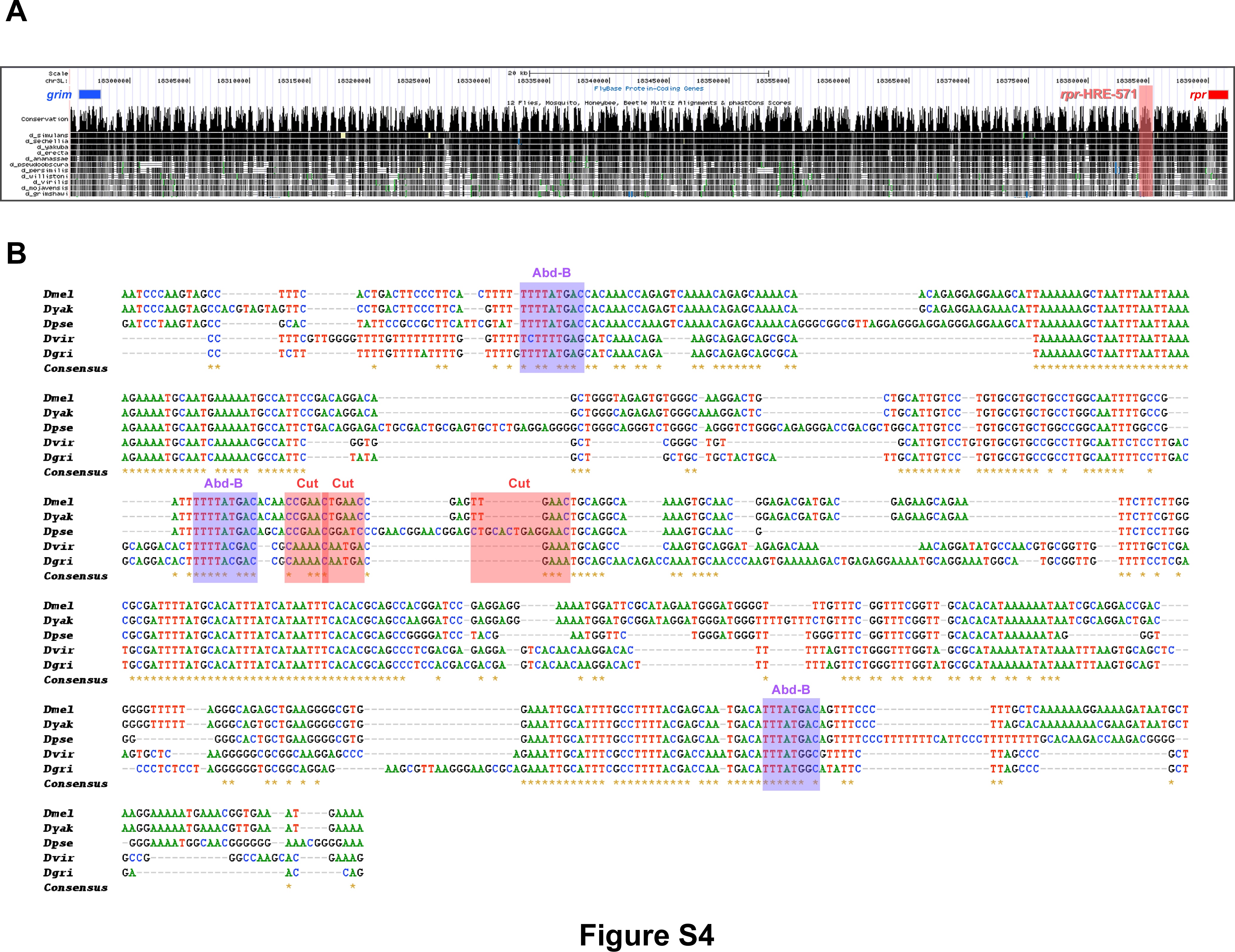

Supplement: Figure S4 — Location and conservation of the rpr-HRE-571 element. (A) Conservation graph of the rpr intergenic region obtained from the UCSC genome browser (http://genome.ucsc.edu/). The rpr-HRE-571 element, which is marked by a light-red box (3 L: 18384438..18385008), is located 6 kb downstream of the rpr coding sequence (marked by a dark-red box). The coding region of the pro-apoptotic gene grim is marked by a dark-blue box. (B) Alignment of the rpr-HRE-571 region from five different Drosophila species (D. melanogaster, D. yakuba, D. pseudoobscura, D. virilis, D. grimshawi). Abd-B binding sites are marked by purple, Ct binding sites by orange boxes. (JPG) [file pgen.1002582.s004.jpg]

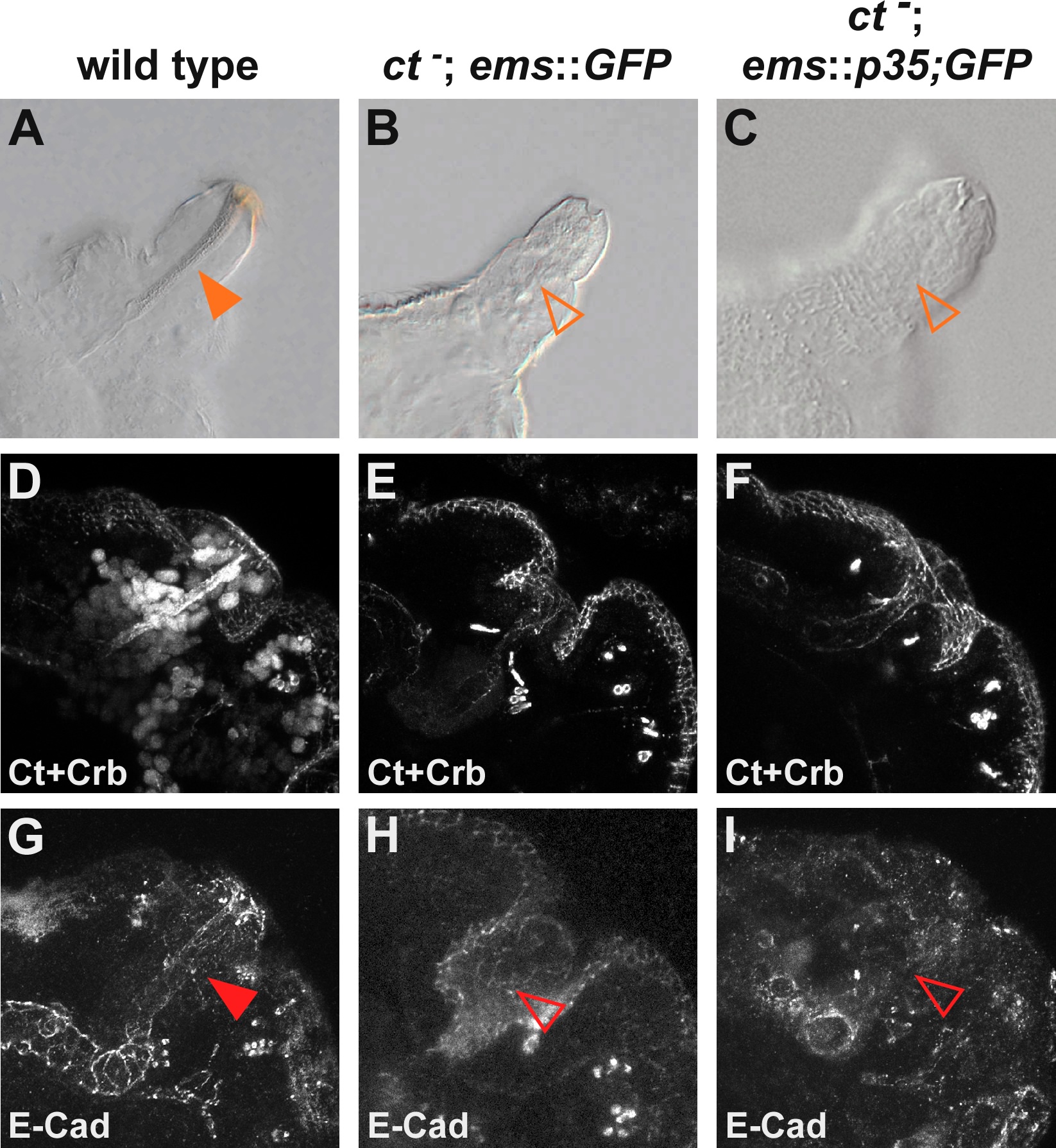

Supplement: Figure S5 — Ct function is required for filzkörper differentiation. The following genotypes are shown: wild type (A, D and G), ctdb7; ems::GFP (B, E and H) and ctdb7; ems::p35;GFP (C, F and I). (A–C) Cuticle preparations of the different genotypes with focus on the posterior spiracle of 1st instar Drosophila larvae. Closed, orange arrowhead in (A) marks the filzkörper, whereas open, orange arrowheads in (B) and (C) indicate the absence of this structure in the respective genotypes. (D–F) Ct and Crb stainings in the respective embryos are shown to highlight the morphology of the filzkörper in the different genotypes. (G–I) DE-Cad staining in the respective genotypes. Closed, red arrowheads in (G) indicate the presence of the filzkörper, whereas open, red arrowheads in (H) and (I) highlight the absence of this structure in ctdb7; ems::GFP (H) and ctdb7; ems::p35;GFP (I) embryos. (JPG) [file pgen.1002582.s005.jpg]

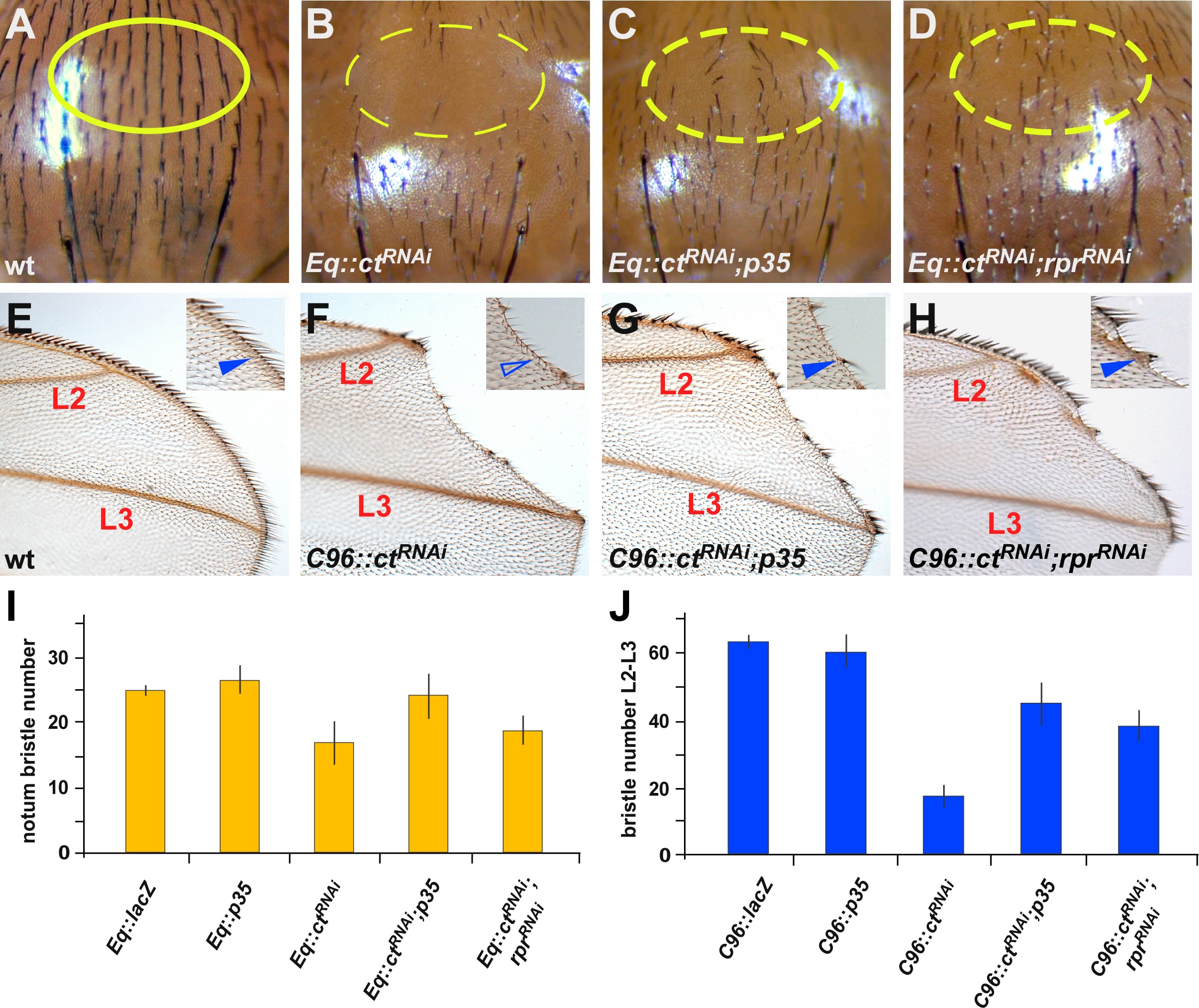

Supplement: Figure S6 — Ct represses apoptosis in wing margin bristles and in external sensory organs of the notum. (A–D) Close-up of Drosophila notum in wild-type (A), Eq::ctRNAi (B), Eq::ctRNAi; p35 (C) and in Eq::ctRNAi; rprRNAi animals (D). Open, yellow circles in (B, C and D) mark the absence of external sensory organs, whereas closed, yellow circle in (A) highlights the presence of these structures in the different genotypes. Note that in (B) most bristles are missing, whereas in (C) and (D) some bristles form, which have cell polarity defects. Expression of Cut in mechanosensory organs of the notum has been shown before [63]. (E–H) Close-up of Drosophila adult wing with focus on wing margin between the wing veins L2 and L3 in wild-type (E), C96::ctRNAi (F), C96::ctRNAi; p35 (G) and in C96::ctRNAi; rprRNAi (H) animals. Closed, blue arrowheads in (E), (G) and (H) highlight the presence of mechanosensory bristles at the wing margin, whereas the open, blue arrowhead in (F) marks their absence in the respective genotype. Importance of Cut function for wing margin development has been shown before [64], [65], [66], [67]. Despite the fact that Cut is expressed in a narrow region along the wing margin [64], [65], [66], [67], we observed a loss of cells outside that region. One likely explanation for this phenotype is the known requirement of Cut to maintain expression of the secreted factor Wingless (Wg) at the wing margin [64], [65], [66], [67], thus we assume that neighboring cells which normally receive the Wg signal undergo apoptosis in a cell non-autonomous manner. (I, J) Quantification of mechanosensory bristles on notum (I) and between wing veins L2 and L3 (J) in the different genetic backgrounds. 15–20 flies were scored for each genotype. (JPG) [file pgen.1002582.s006.jpg]

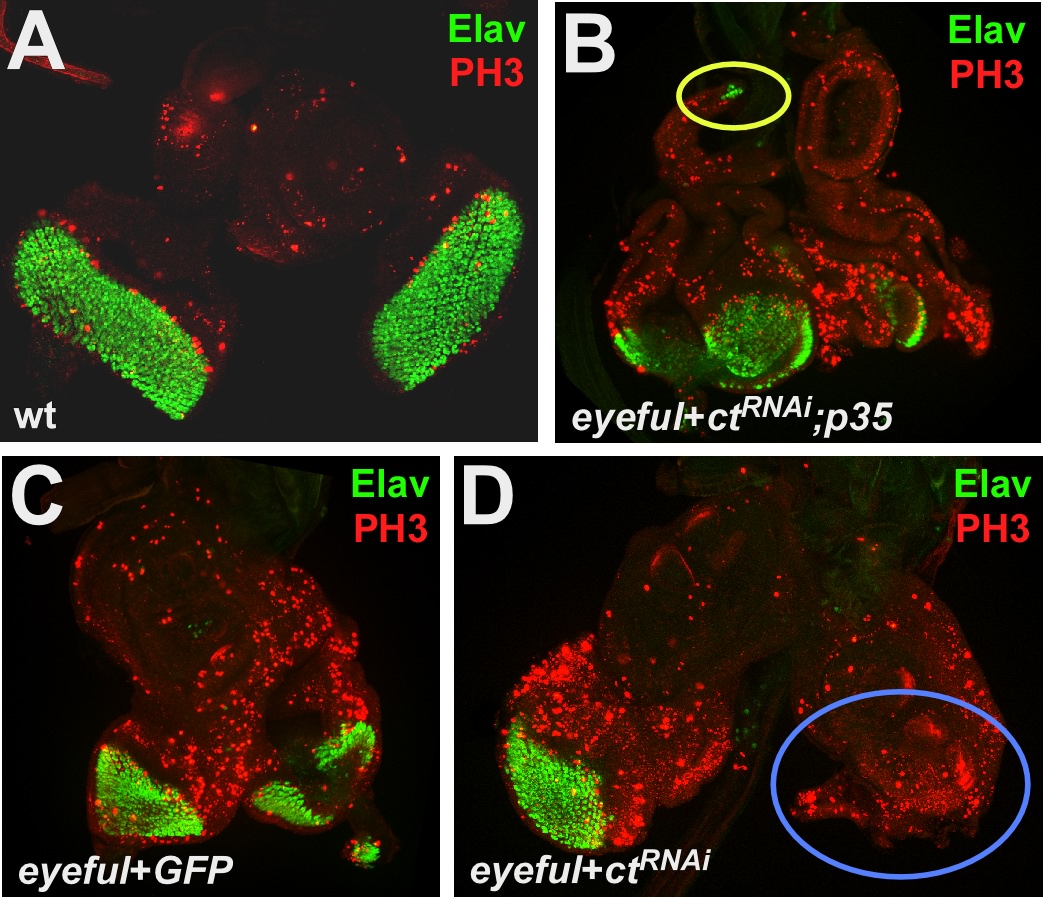

Supplement: Figure S7 — Invasiveness of Ct depleted cells. (A–D) Co-localization of the eye differentiation marker Embryonic Lethal Abnormal Vision (ELAV) and the proliferation marker Phosphorylated histone H3 (PH3) in 3rd instar eye-antennal discs. Blue circle in (D) marks loss of ELAV expression in eyeful::ctRNAi 3rd instar eye-antennal discs, yellow circle in (B) marks ELAV-positive cells at ectopic location in eyeful::ctRNAi;p35 3rd instar eye-antennal discs. (JPG) [file pgen.1002582.s007.jpg]

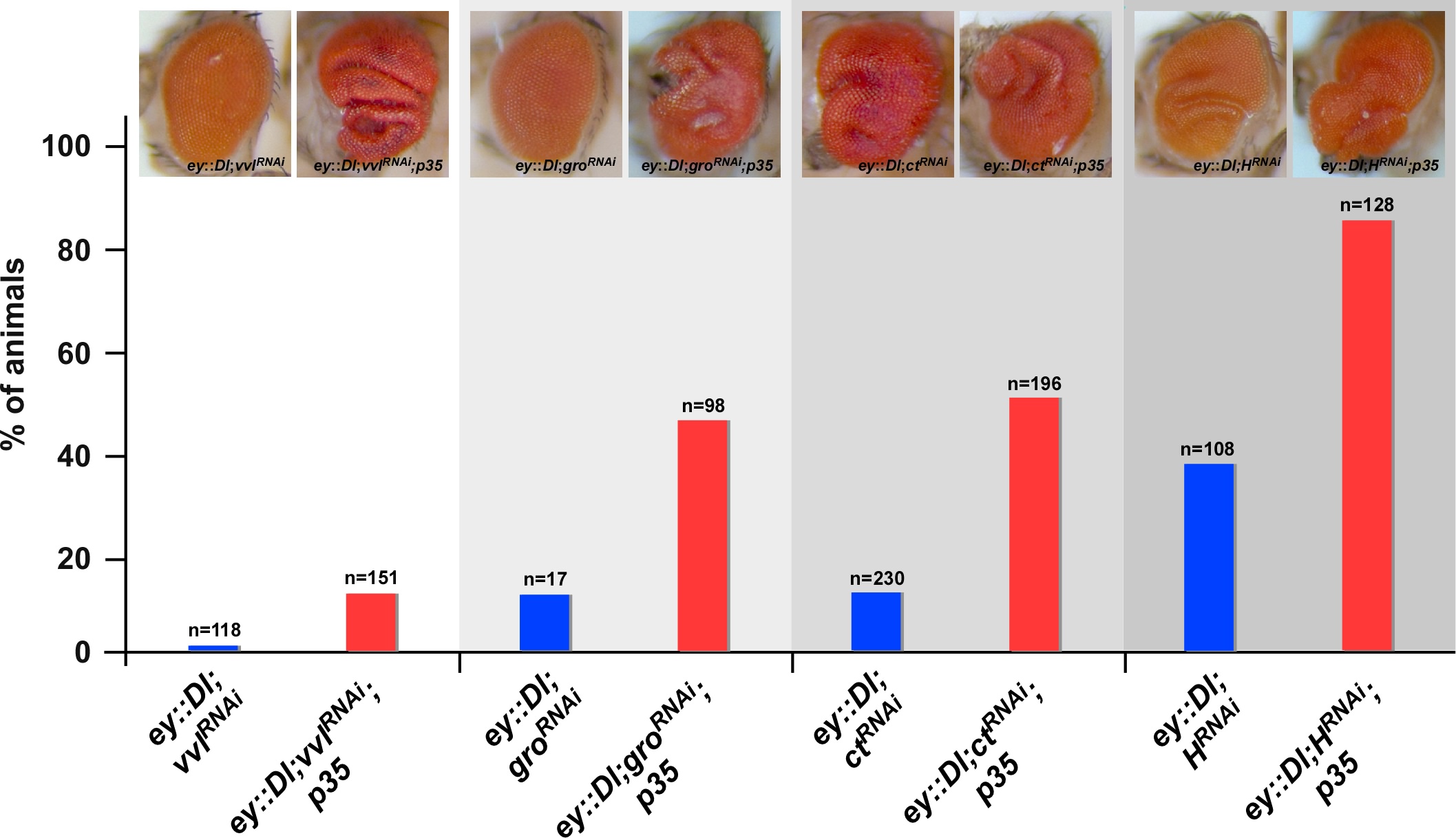

Supplement: Figure S8 — Simultaneous regulation of differentiation and apoptosis represents a general cancer prevention mechanism. Top: Representative pictures of tumorous eye growth in flies of indicated genotypes. Bottom: Quantification of primary tumor growth in the respective genotypes. Genes tested were selected based on their function as cell-type specifying transcriptional regulators active in the Drosophila eye. Genes: vvl: ventral veins lacking; gro: groucho; ct: cut; H: Hairless. (JPG) [file pgen.1002582.s008.jpg]
