# Supplementary material for: Antagonistic Regulation of Apoptosis and Differentiation by the Cut Transcription Factor Represents a Tumor-Suppressing Mechanism in Drosophila
Source: PLoS Genet. 2012 Mar 15;8(3):e1002582. doi: 10.1371/journal.pgen.1002582 (PMC3305397; doi:10.1371/journal.pgen.1002582)
Supplement: Table S1 — Putative binding sites for vertebrate Cux1 within the non-coding regions of the puma gene. (DOC) [file pgen.1002582.s009.doc]

| **Gene** | **Source** | **Region** | **Cux binding site #** |
| --- | --- | --- | --- |
| *BBC3* (*puma*, Ensembl: chr19:47724079..47736023 | *Homo sapiens* Ensembl | Upstream Intergenic  chr19:47713893..47724078 | 101 |
|  |  | Downstream Intergenic  chr19:47736023..47759730 | 196 |
|  |  | Intron 1 | 18 |
|  |  | Intron 2 | 6 |
|  |  | Intron 3 | 44 |
